# The microbiota in bronchoalveolar lavage from young children with chronic lung disease includes taxa present in both the oropharynx and nasopharynx

**DOI:** 10.1186/s40168-016-0182-1

**Published:** 2016-07-07

**Authors:** R. L. Marsh, M. Kaestli, A. B. Chang, M. J. Binks, C. E. Pope, L. R. Hoffman, H. C. Smith-Vaughan

**Affiliations:** Menzies School of Health Research, Charles Darwin University, PO Box 41096, Casuarina, Darwin, NT 0810 Australia; Research Institute for the Environment and Livelihoods, Charles Darwin University, Darwin, NT Australia; Queensland Children’s Medical Research Institute, Queensland University of Technology, Brisbane, QLD Australia; Department of Pediatrics, University of Washington, Seattle, WA USA; Department of Microbiology, University of Washington, Seattle, WA USA; School of Medicine, Griffith University, Gold Coast, QLD Australia

**Keywords:** Paediatric lung disease, Upper airways, Lower airways, Oropharynx, Nasopharynx, Bronchoalveolar lavage, Microbiota

## Abstract

**Background:**

Invasive methods requiring general anaesthesia are needed to sample the lung microbiota in young children who do not expectorate. This poses substantial challenges to longitudinal study of paediatric airway microbiota. Non-invasive upper airway sampling is an alternative method for monitoring airway microbiota; however, there are limited data describing the relationship of such results with lung microbiota in young children. In this study, we compared the upper and lower airway microbiota in young children to determine whether non-invasive upper airway sampling procedures provide a reliable measure of either lung microbiota or clinically defined differences.

**Results:**

The microbiota in oropharyngeal (OP) swabs, nasopharyngeal (NP) swabs and bronchoalveolar lavage (BAL) from 78 children (median age 2.2 years) with and without lung disease were characterised using 16S rRNA gene sequencing. Permutational multivariate analysis of variance (PERMANOVA) detected significant differences between the microbiota in BAL and those in both OP swabs (*p* = 0.0001, Pseudo-*F* = 12.2, *df* = 1) and NP swabs (*p* = 0.0001; Pseudo-*F* = 21.9, *df* = 1) with the NP and BAL microbiota more different than the OP and BAL, as indicated by a higher Pseudo-*F* value. The microbiota in combined OP and NP data (upper airways) provided a more comprehensive representation of BAL microbiota, but significant differences between the upper airway and BAL microbiota remained, albeit with a considerably smaller Pseudo-*F* (PERMANOVA *p* = 0.0001; Pseudo-*F* = 4.9, *df* = 1). Despite this overall difference, paired BAL and upper airway (OP and NP) microbiota were >50 % similar among 69 % of children. Furthermore, canonical analysis of principal coordinates (CAP analysis) detected significant differences between the microbiota from clinically defined groups when analysing either BAL (eigenvalues >0.8; misclassification rate 26.5 %) or the combined OP and NP data (eigenvalues >0.8; misclassification rate 12.2 %).

**Conclusions:**

Upper airway sampling provided an imperfect, but reliable, representation of the BAL microbiota for most children in this study. We recommend inclusion of both OP and NP specimens when non-invasive upper airway sampling is needed to assess airway microbiota in young children who do not expectorate. The results of the CAP analysis suggest lower and upper airway microbiota profiles may differentiate children with chronic suppurative lung disease from those with persistent bacterial bronchitis; however, further research is needed to confirm this observation.

**Electronic supplementary material:**

The online version of this article (doi:10.1186/s40168-016-0182-1) contains supplementary material, which is available to authorized users.

## Background

In recent years, molecular methods have clearly demonstrated that the lungs are not sterile at homeostasis. Adult studies have shown that microaspiration of the upper respiratory secretions continually inoculates the lungs with oral microbiota [1]; host defences must then efficiently clear or contain the microbes to prevent infection [2]. Changes in the lower airway microbiota during the onset and progression of acute and chronic lung diseases are poorly understood.

In adults and older children, spontaneous and induced sputum are used non-invasively to sample the lower airways for assessment of the lung microbiota. Studies in very young children who do not expectorate are more challenging as invasive procedures, such as bronchoalveolar lavage (BAL) collected via bronchoscopy, are required to sample the lower airway microbiota [3]. The invasive nature of BAL, including a requirement for general anaesthesia, renders it neither suitable nor ethically feasible for longitudinal studies [4]. Reflecting this limitation, studies of lung microbiota in very young children have generally been cross-sectional [5].

Upper airway sampling has long been considered an alternative method for assessing airway microbiology in young children [6] but remains controversial as significant differences between the upper and lower airway microbiota have been detected in several studies [7–11]. In recent years, studies comparing upper and lower airway specimens have reported that the lower airway microbiota is more similar to that of the oropharynx than that in the nose or nasopharynx [1, 12, 13]; however, these studies have been limited to adults [1, 12] and older children (>7 years) [13]. It is unclear if these data can be extrapolated to other paediatric populations, especially as the nasopharynx is an important reservoir of lower respiratory pathogens in young children [14, 15]. Numerous studies have highlighted the importance of the nasopharyngeal (NP) microbiota, particularly pathogenic species, to lung disease in infants and young children [16–21]. Furthermore, culture-based studies have demonstrated that oropharyngeal (OP) swabs do not reliably predict the presence of respiratory pathogens in the lower airways of children <2 years of age [6].

In this study, we have directly compared OP, NP and BAL microbiota in young children. Our study included 78 children who had protracted bacterial bronchitis (PBB), chronic suppurative lung disease (CSLD) or no lung disease (controls). We aimed to determine (i) the similarity of the microbiota in the OP and NP compared to BAL and (ii) whether non-invasive sampling of the upper airways provided a reliable measure of either the lung microbiota or differences between clinically defined groups. We hypothesised that the BAL microbiota in young children would show similarity to both the OP and NP microbiota.

## Results

The study cohort (Table 1) included 78 children—40 children with CSLD, including 36 with idiopathic bronchiectasis confirmed by chest high-resolution computed tomography (cHRCT), 28 with PBB and 10 controls. The median age of the children in the three groups were similar (median 2.2 years; Kruskal-Wallis *p* = 0.19, Table 1). Twenty percent of the control children, 21 % of the children with PBB and 65 % of the children with CSLD had recently received antibiotics (defined as antibiotic treatment within 2 weeks of bronchoscopy). Eighty-three percent of children with CSLD, 7 % of children with PBB and none of the controls were Indigenous Australians (Table 1).

**Table 1:** Study cohort

	Controls	PBB	CSLD
Number of children	10	28	40 (36 with BE)
Region			
Brisbane	10	28	5 (all with BE)
Darwin	0	0	35 (31 with BE)
Median age in years (95 % CI)	2.0 (1.0–4.0)	1.8 (1.4–2.3)	2.6 (2.1–3.1)
Number of Indigenous children (%)	0 (0)	2 (7)	33 (83)
Males (%)	8 (80)	16 (57)	23 (58)
Received antibiotics within 2 weeks of bronchoscopy (%)	2 (20)	6 (21)	26 (65)
Azithromycin	1	1	17
Amoxicillin	0	0	1
Amoxicillin clavulanate	1	3	1
Sulfamethoxazole trimethoprim	0	2	1
Roxithromycin	0	0	1
Ceftriaxone	0	0	2
Azithromycin + sulphamethoxazole trimethoprim	0	0	1
Ceftriaxone, amoxicillin clavulanate, flucloxicillin	0	0	1
Unsure if received antibiotics	0	0	1

*BE* idiopathic bronchiectasis

**Fig. 1:**
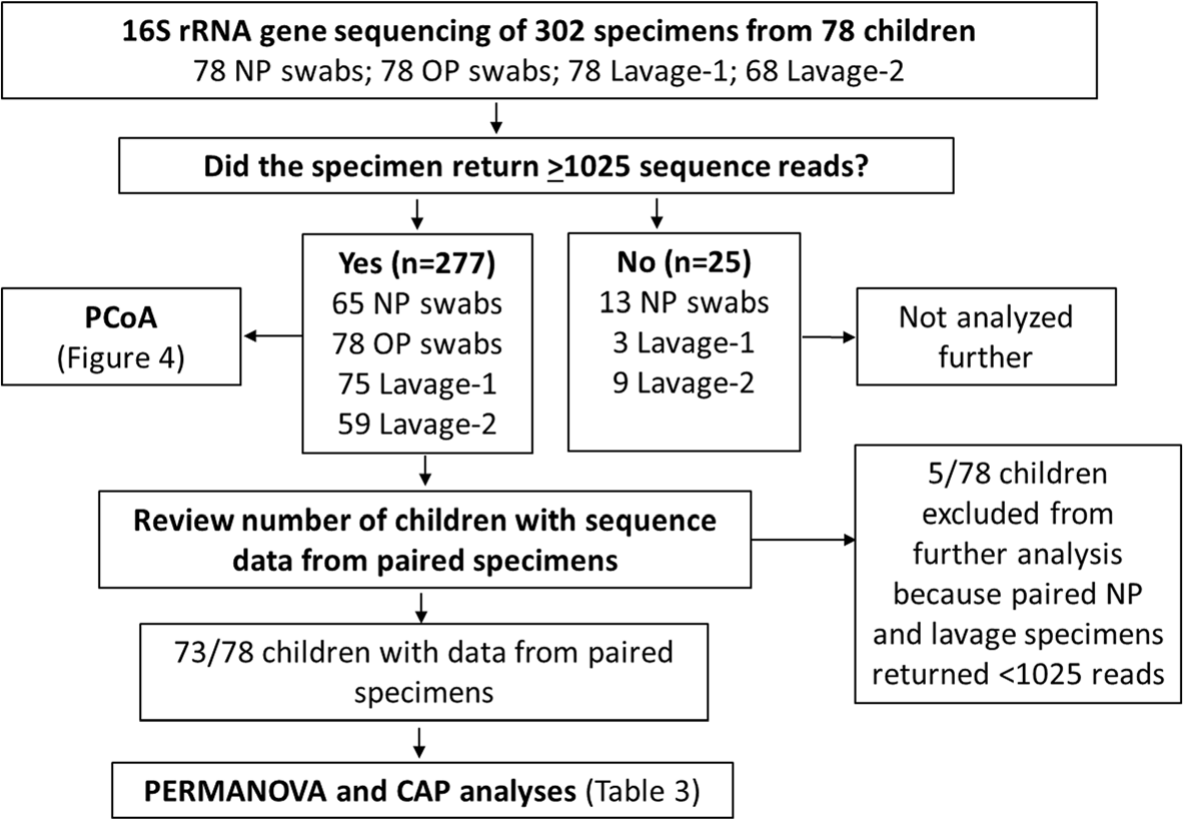
Summary of specimen processing after 16S rRNA gene sequencing. Specimens that returned <1025 reads were excluded from further analysis as bacterial community coverage in these specimens (based on rarefaction and Good’s coverage) was too low

An OP swab, NP swab and two sequential BAL specimens (Lavage-1 and Lavage-2) were available for 68 children (36 with CSLD, 22 with PBB and 10 control children). For the remaining 10 children, only the OP swab, NP swab and Lavage-1 were available. Thus, 302 specimens from 78 children were available for testing (Fig. 1).

### DNA extracts from a high proportion of clinical specimens reflected low bacterial load

**Fig. 2:**
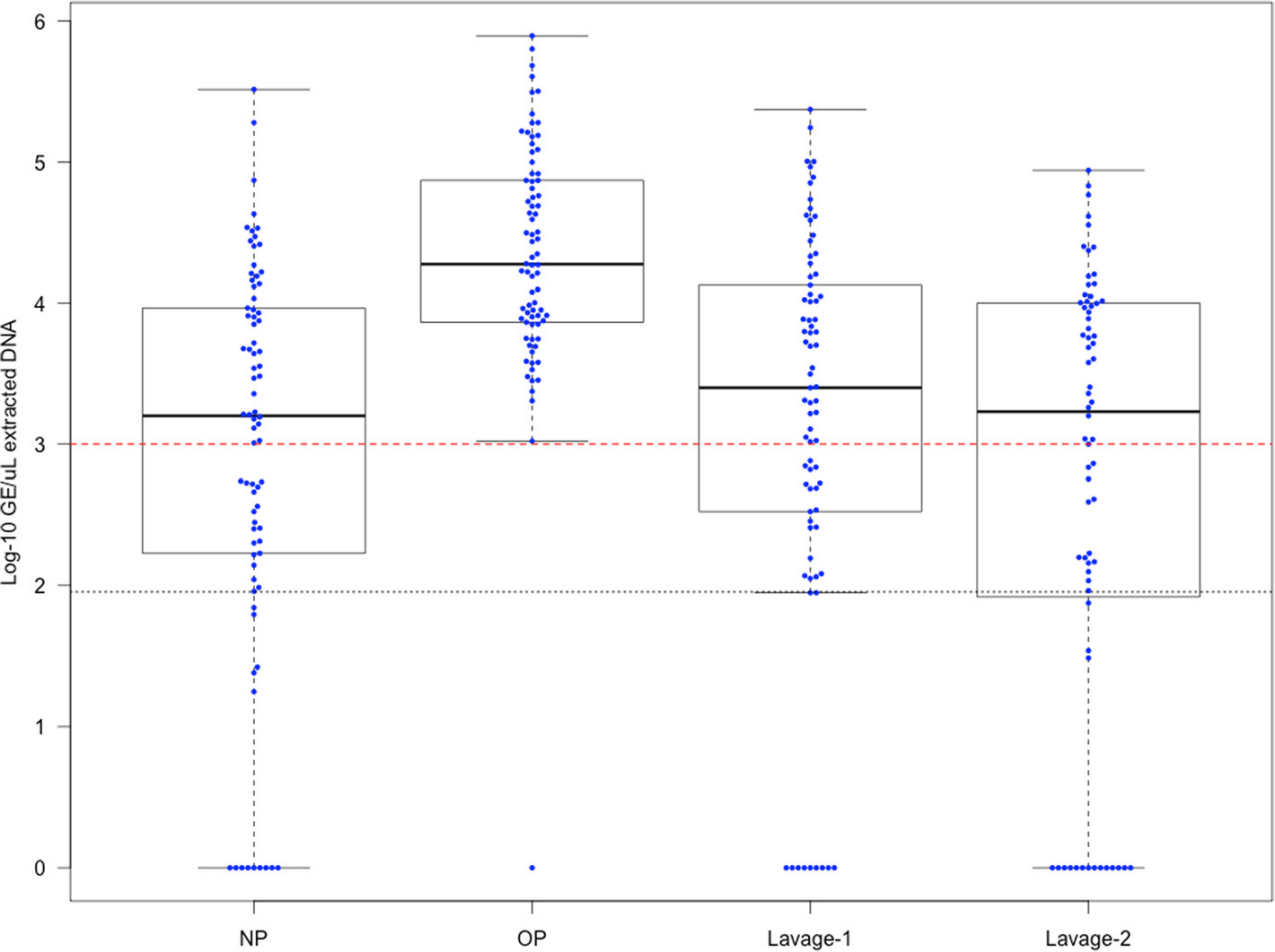
Bacterial load in DNA extracted from clinical specimens. Bacterial load is expressed on a log-scale as genome equivalents (GE)/μL extracted DNA. The *red dashed line* indicates 1 × 10^3^ GE/μL of the extracted DNA. The *black dotted line* indicates the qPCR limit of detection (90 GE). Quantification below the qPCR limit of detection (90 GE) is unreliable. *NP* NP swab, *OP* OP swab

The bacterial load in each DNA extract was estimated by qPCR [22, 23]. A level of >10^3^ genome equivalents (GE)/μL extracted DNA is recommended for 16S rRNA gene sequencing [24, 25]. This threshold was met by 207/302 (68 %) specimens. Bacterial load could not be estimated for one Lavage-1 sample due to the presence of PCR inhibitors. Low bacterial load (defined here as <10^3^ GE/μL extracted DNA) was detected for 33/78 (42 %) NP swabs, 1/78 (1.3 %) OP swabs, 29/77 (38 %) Lavage-1 and 31/68 (46 %) Lavage-2 specimens (Fig. 2); 44 of these specimens failed to amplify above the qPCR limit of detection (90 GE), indicating very low bacterial load or an absence of bacteria (15 NP swabs, 1 OP swab, 11 Lavage-1 and 17 Lavage-2 specimens; Fig. 2).

The data were then reviewed to determine if specimens with low bacterial loads were provided by the same or by different children. Overall, low bacterial load was detected in at least one specimen from 49/78 (63 %) children. Approximately half of these children (49 %) had recently received antibiotics, a proportion similar to the antibiotic exposure rate for all subjects (43 %), suggesting that low bacterial load was unrelated to recent antibiotic exposure. DNA extracts from all of the specimens from one control child had low bacterial load; this child had received amoxicillin clavulanate. A further 14 children had low bacterial loads detected for the NP swab, Lavage-1 and Lavage-2 specimens (4 control children, 1 child with PBB and 9 with CSLD); six of these children had recently received antibiotics. Low bacterial load was detected in the NP swabs from 17 children from whom the paired Lavage-1 specimens contained >10^3^ GE/μL extracted DNA. No children had low bacterial load in the OP swab when the load in paired Lavage-1 specimen was >10^3^ GE/μL extracted DNA. Bacterial load in any specimen type was unrelated to the age of the children (all Spearman rank correlation *p* > 0.29).

### 16S rRNA gene sequencing was successful for most specimens, including those with low bacterial load

Regardless of the bacterial load, all of the 302 specimens were processed for 16S rRNA gene sequencing. One NP swab, two Lavage-1 specimens and one Lavage-2 specimen failed to generate any 16S rRNA gene sequencing data; each of these specimens also failed to amplify in the bacterial load qPCR.

As a high proportion of the specimens had low bacterial load, the sequence data were reviewed for the presence of probable contaminant taxa [24, 26], as described in Additional file 1. Overall, 237 operational taxonomic units (OTUs) consistent with 54 genera reported previously as contaminants in DNA extraction reagents [24, 26] were identified and removed prior to downstream analyses (Additional file 1: Table S1). The relative abundance of these OTUs ranged from 0 to 89 % (median 0.2 %) and showed a significant inverse correlation with the bacterial load in the extracted DNA (Spearman rho = −0.85; *p* < 0.0001). All specimens with probable contaminant genera detected at relative abundance >20 % had low bacterial load. There was no significant difference in the relative abundance of the probable contaminant OTUs in NP swabs, Lavage-1 or Lavage-2 specimens (Additional file 1: Figure S1). The relative abundance of the probable contaminant OTUs did not exceed 20 % in any OP swab, reflecting the high proportion of OP swabs (77/78) that had bacterial load >10^3^ GE/μL extracted DNA (Fig. 2).

After exclusion of probable contaminant OTUs, there were 90–17,444 sequencing reads/specimen (median 4639). The data were subsampled to 1025 reads/specimen to achieve a balance between sufficient sampling depth and exclusion of low-quality specimens. Good’s coverage at this depth was >95 % for the 277 specimens with >1025 reads. This included 77/94 (82 %) specimens that had low bacterial load, 27 of which had failed to amplify above the qPCR’s limit of detection. After removal of the contaminant OTUs, low bacterial load specimens did not cluster separately to specimens with >10^3^ GE/μL extracted DNA in principal coordinate analysis (PCoA; Additional file 1: Figure S2).

Twenty-five specimens had <1025 reads and were excluded from further analysis (Fig. 1). This left 277 specimens from 78 children. For 73 children, paired sequence data were available from the upper and/or lower airway specimens. Of these, 65/73 children had sequence data for the OP swab, NP swab and Lavage-1 (8 controls, 19 with PBB and 38 with CSLD); 57/78 had data for Lavage-1 and Lavage-2 (6 controls, 19 PBB and 32 with CSLD); and 49 had data for all four specimen types (5 controls, 13 with PBB and 31 with CSLD). Five children were excluded from analyses of paired data because their NP swab and one or both lavage specimens returned <1025 sequence reads (Fig. 1).

### Alpha diversity in the OP, NP and lower airways

**Fig. 3:**
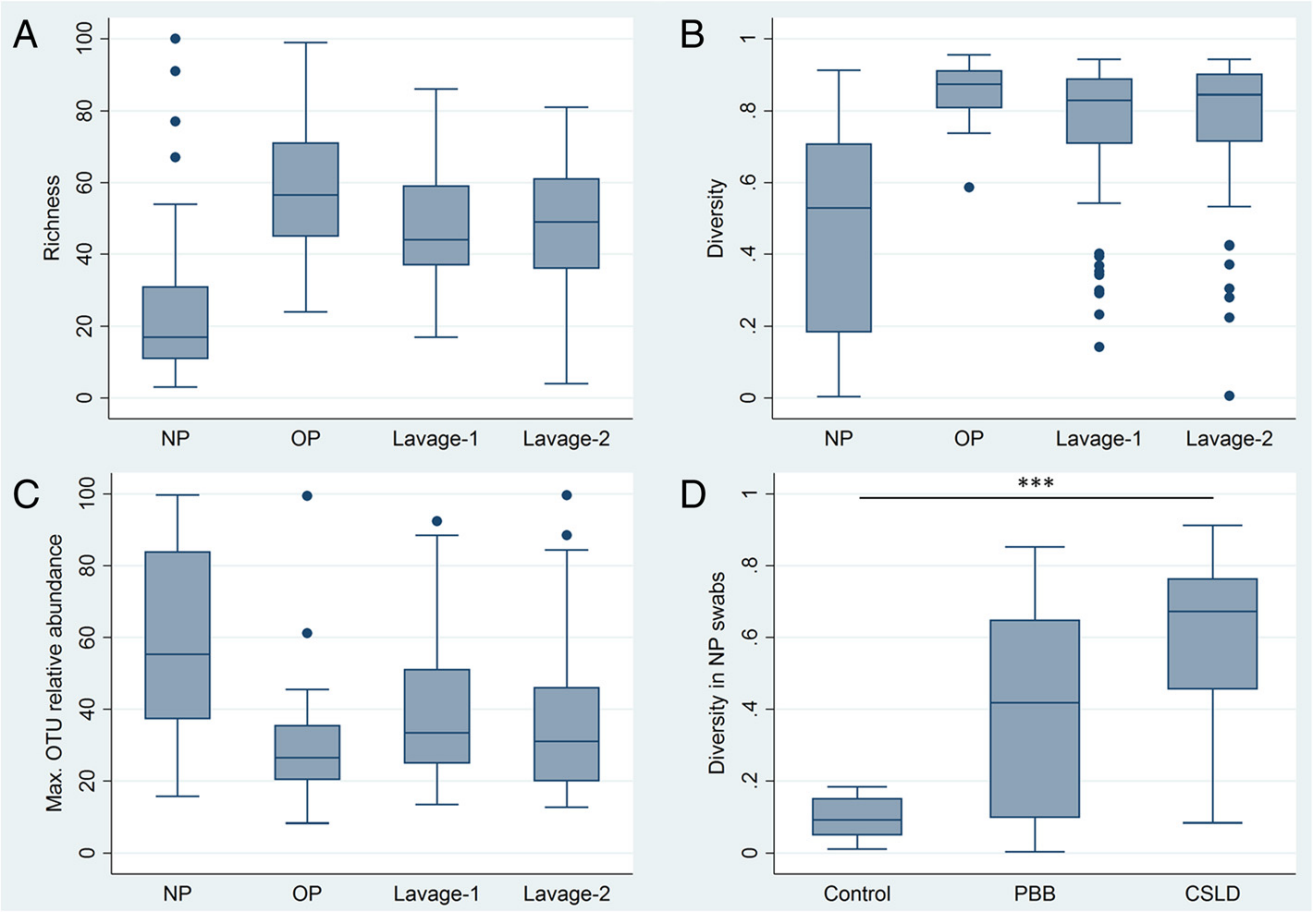
Alpha diversity in the microbiota of different specimen types. **a** OTU-level richness in different specimen types. **b** Simpson’s index of diversity in different specimen types. Values approaching one indicate higher richness and more even community structure. Values approaching zero indicate communities dominated by a small number of taxa. **c** Relative abundance of the most dominant OTU in different specimen types (Max. OTU relative abundance). **d** Diversity in NP swabs from each diagnostic group. Diversity in NP swabs from control children was significantly lower than that in children with CSLD (Dunn’s post hoc test, *p* < 0.0001); comparisons between other groups did not reach statistical significance after correcting for multiple measures. *NP* NP swabs, *OP* OP swabs

A total of 1954 OTUs were present in the subsampled dataset, with 290 (14.8 %) OTUs present at >1 % relative abundance. Bacterial richness ranged from 3 to 100 OTUs per specimen (Fig. 3a). Simpson’s index of diversity ranged from 0.003 to 0.955 (Fig. 3b). Diversity by Simpson’s index was significantly lower in NP swabs compared to all other specimen types (all Dunn’s post hoc test, *p* < 0.0001), reflecting the more common dominance of individual OTUs in the NP microbiota (Fig. 3c). Overall, 69.2 % of NP swabs, 1.3 % OP swabs, 25 % Lavage-1 and 22 % Lavage-2 specimens were dominated by a single OTU at >50 % relative abundance. Dominant OTUs were consistent with taxa commonly detected in airway specimens, including *Moraxella*, *Haemophilus*, *Staphylococcus*, *Streptococcus*, *Neisseria*, *Prevotella* and *Corynebacterium* species (Table 2).

**Table 2:** OTUs detected at >50 % relative abundance in each specimen type

Specimen type	OTU	SILVA reference taxonomy	BLASTn (% identity)	Maximum relative abundance (%)
NP swabs	OTU00003	Moraxella	*Moraxella catarrhalis* (98 %)	99.8
	OTU00002	Pasteurellaceae	*Haemophilus influenzae* (99 %)	96.7
	OTU00008	Staphylococcus	*Staphylococcus aureus* (100 %)	96.4
	OTU00012	Corynebacterium	*Corynebacterium propinquum* (100 %)	98.1
	OTU00001	Streptococcus	Mitis Group Streptococci (99 %)	94.9
	OTU00022	Moraxella	*Moraxella nonliquefaciens* (99 %)	94.1
	OTU00017	Enhydrobacter	*Moraxella lincolnii* (98 %)	81.2
	OTU00061	Unclassified	*Mycoplasma amphoriforme* (99 %)	61.3
	OTU00029	Flavobacteriaceae	*Ornithobacterium rhinotracheale* (90 %)	54.7
	OTU00060	Proteobacteria	Unclassified	53.6
OP swabs	OTU00006	Bacteroidetes	*Porphyromonas* sp. (99 %)	62.5
Lavage-1	OTU00003	Moraxella	*Moraxella catarrhalis* (98 %)	92.6
	OTU00008	Staphylococcus	*Staphylococcus aureus* (100 %)	83.8
	OTU00002	Pasteurellaceae	*Haemophilus influenzae* (99 %)	83.5
	OTU00022	Moraxella	*Moraxella nonliquefaciens* (99 %)	79.2
	OTU00011	Neisseria	*Neisseria lactamica* (99 %)	60.2
	OTU00004	Prevotella	*Prevotella* sp. (98 %)	51.9
Lavage-2	OTU00002	Pasteurellaceae	*Haemophilus influenzae* (99 %)	99.7
	OTU00003	Moraxella	*Moraxella catarrhalis* (98 %)	87.7
	OTU00001	Streptococcus	Mitis Group Streptococci (99 %)	67.4
	OTU00004	Prevotella	*Prevotella* sp. (98 %)	51.2

BLASTn identities were selected based on 100 % coverage and >98 % identity score (% identity), except for OTU00029 which was not identified above 90 % identity. This OTU was conditionally identified as *Ornithobacterium rhinotracheale*, but this taxonomy must be interpreted with caution. Bacterial load in specimens dominated by the Enhydrobacter OTU at >50 % relative abundance was consistently >1 × 10^3^ GE/μL extracted DNA (Additional file 1: Figure S5)

Diversity in any specimen type was not significantly associated with antibiotic treatment within 2 weeks of bronchoscopy and was unrelated to bacterial load in OP swab, NP swab and Lavage-2 specimens (all Mann-Whitney *U* test *p* > 0.05). Diversity was significantly higher in Lavage-1 specimens with low bacterial load (median 0.89; 95 % CI 0.83–0.91) compared to those with higher loads (median 0.79, 95 % CI 0.71–0.83; Mann-Whitney *U* test *p* = 0.0005); however, this finding is unlikely to indicate a systemic bias as it was not reproduced in analysis of other specimen types. Diversity in any specimen type was unrelated to the age of the children (all Spearman correlation *p* > 0.08).

Diversity in NP swabs increased with disease severity (Fig. 3d); however, only the comparison of the control and CSLD children showed significant differences after correction for multiple measures (Dunn’s post hoc test, *p* < 0.0001). There was no significant difference in the microbiota diversity in the OP, Lavage-1 and Lavage-2 specimens from different diagnostic groups (Additional file 1: Figure S3). There was no significant correlation between the diversity in lavage specimens and that in corresponding OP or NP swabs (all Spearman rank correlation *p* > 0.07), reflecting differences in the relative abundance of dominant OTUs in the upper and lower airway data.

### Comparison of OP and NP microbiota

**Fig. 4:**
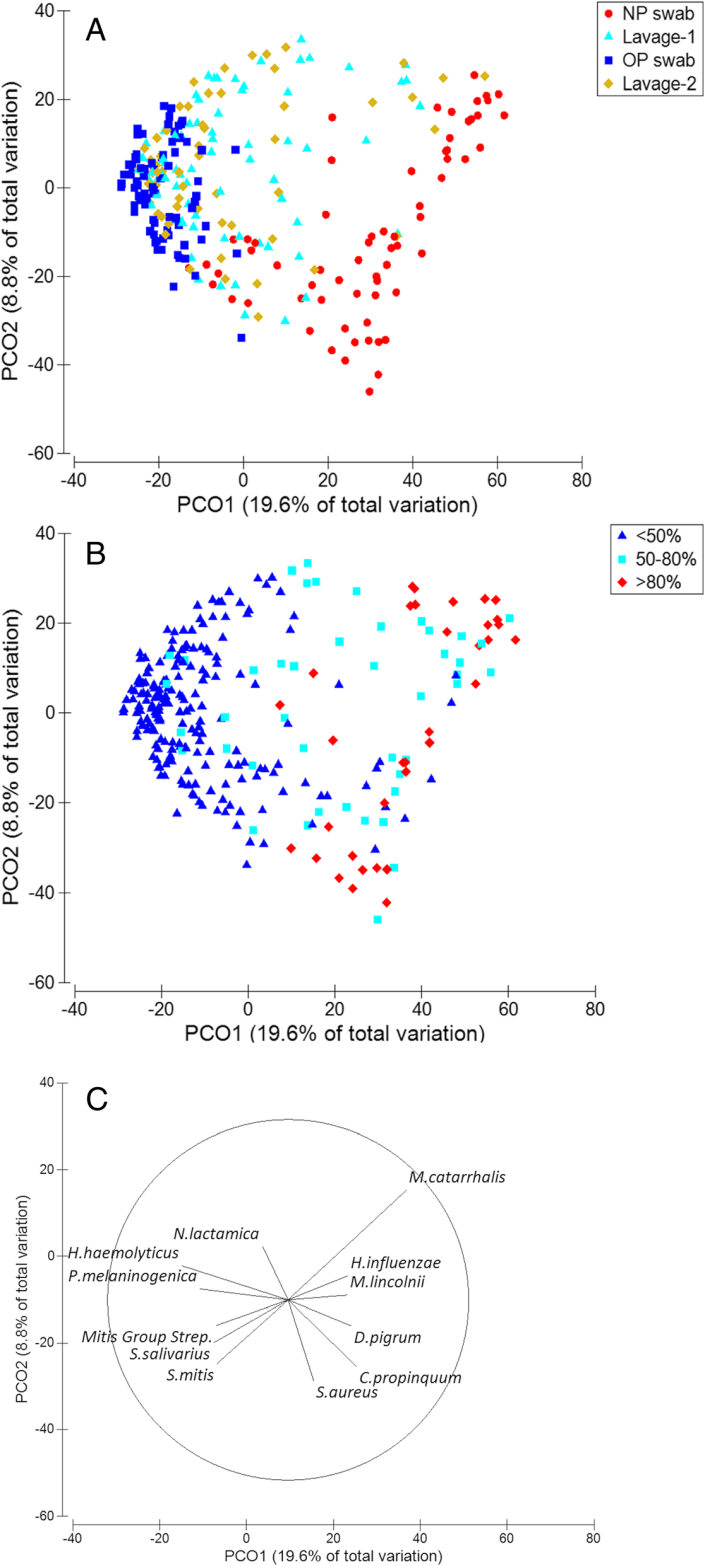
Lavage microbiota shows similarity to both OP and NP. Principal coordinate analysis (PCoA) demonstrating relationships between the microbiota in different specimen types based on a Bray-Curtis similarity matrix derived from square root transformed OTU-level data. **a** The NP and OP microbiota were distinct, whereas Lavage-1 and Lavage-2 specimens were dispersed between the OP and NP data points. *Red circles* = NP swabs. *Dark blue squares* = OP swabs. *Light blue triangles* = Lavage-1. *Mustard diamonds* = Lavage-2. **b** Dispersion across the main axis (PCO1) reflected differences in the relative abundance of the dominant OTU. The PCoA is identical to that in part A of this figure but has been coloured to indicate the relative abundance of the dominant OTU. *Dark blue triangles* = dominant OTU at <50 % relative abundance. *Light blue squares* = dominant OTU at 50–80 % relative abundance. *Red circles* = dominant OTU at >80 % relative abundance. **c** A vector plot visualising directional effects of dominant OTUs (Table 2) in the different specimen types. Vectors show the Pearson rank correlation between each OTU and the PCO axes. The vectors are labelled to indicate the BLAST identification of dominant OTUs. The Mitis Group Streptococci vector indicates a single OTU that could not be identified to the species level. Mitis Group Streptococci include *Streptococcus pneumoniae*. Vectors are not shown for *Porphyromonas*, *Terrahaemophilus*, and *Gemella* OTUs as they overlapped with *Neisseria*, *Prevotella* and *Haemophilus haemolyticus*, respectively. Likewise, vectors are not shown for *Granulicatella adiacens* and *Moraxella nonliquiefaciens* as they overlapped with the *Streptococcus mitis* and *Moraxella lincolnii* vectors, respectively

Consistent with earlier studies [1, 12, 13, 25], the OP and NP microbiota were distinct. Median Bray-Curtis similarity between paired OP and NP swabs was only 10.1 % (95 % CI 8.2–12.6). In a PCoA (Fig. 4a), OP and NP data points were well separated across the first axis (PCO1), which explained 19.6 % of the total data variability. Separation across PCO1 also reflected differences in the relative abundance of the dominant OTU in each specimen (Fig. 4b). A vector plot showing directional effects for the most dominant OTUs was consistent with separation of OP and NP microbiota across PCO1 (Fig. 4c). A two-way crossed permutational multivariate analysis of variance (PERMANOVA) with specimen type as a fixed factor and the children as a random factor confirmed that the microbiota were significantly different between the OP and NP specimens (*p* = 0.0001, Pseudo-*F* 35.3, degrees of freedom (*df*) 1), with 35.5 % of the total variation explained by differences between the two specimen types. This variation was 2.3 times larger than the variation between children (15.7 %; Table 3).

**Table 3:** Similarity of the upper and lower airway microbiota

Comparison	Number of children	Bray-Curtis similarity median (95 % CI)	PERMANOVA						
			Variation between specimen types			Variation between children			Residual variation
			*p*	Pseudo-*F* (*df*)	%CoV	*p*	Pseudo-*F* (*df*)	%CoV	%CoV
NP and OP	65	10.1 (8.2–12.6)	0.0001*	35.3 (1)	35.5	0.001*	1.2 (64)	15.7	48.8
Lavage-1 and Lavage-2	57	70.9 (63.2–74.0)	0.72	0.8 (1)	n/a	0.0001*	6.01 (57)	61.4	38.6
NP and Lavage-1	65	25.6 (22.3–28.8)	0.0001*	21.9 (1)	25.3	0.0001*	1.9 (64)	30.1	44.6
OP and Lavage-1	65	54.5 (44.9–57.6)	0.0001*	12.2 (1)	17.9	0.0001*	2.7 (64)	39.2	42.9
Upper and Lavage-1	65	53.5 (48.4–56.7)	0.0001*	6.8 (1)	13.0	0.0001*	3.0 (64)	43.4	43.5
Upper and lower	49	55.5 (51.7–58.6)	0.0001*	4.9 (1)	12.1	0.0001*	3.2 (48)	45.1	42.8

The microbiota in paired Lavage-1 and Lavage-2 specimens showed high similarity, whereas the microbiota in paired OP and NP swabs were distinct. Lavage-1 microbiota was significantly different to the OP and NP microbiota. The highest similarity between the upper and lower airway data was obtained by combining OP with NP data (to represent the upper airways) and Lavage-1 with Lavage-2 data (to represent the lower airways). All analyses were performed using a Bray-Curtis similarity matrix based on square root transformed OTU-level data. *CoV* is the estimated components of variation and indicates the data variability due to the specified factor. Comparisons were limited to the 73 children whose paired specimens each returned >1025 16S rRNA gene sequence reads (Fig. 1). Lavage-2 data were only available for a subset of children

*n/a* not applicable

*Indicates significant results

### Comparison of Lavage-1 and Lavage-2 microbiota

There was no significant difference between the bacterial loads in paired Lavage-1 and Lavage-2 specimens (Mann-Whitney *U* test *p* = 0.39). Likewise, there was no difference between the microbiota in Lavage-1 and Lavage-2 (PERMANOVA *p* = 0.72, Pseudo-*F* = 0.8, *df* 1; Table 3). Median Bray-Curtis similarity between paired lavage specimens was 70.9 % (95 % CI 63.2–74.0), a level comparable to that of the sequencing reproducibility control (71.1 %; this control is described further in the methods and Additional file 1). In a PCoA, the lavage specimens were dispersed across PCO1 between the OP and NP data points (Fig. 4a), suggesting similarity of the lavage microbiota to both the OP and NP microbiota in some children. The similarity percentages routine (SIMPER) in PRIMER identified three OTUs consistent with Mitis Group Streptococci (including *Streptococcus pneumoniae*), *Haemophilus influenzae* and *Prevotella* as individually accounting for most variation between lavage specimens (16, 11 and 7 %, respectively). The relative abundance of these OTUs in lavage specimens was significantly correlated with that in either the OP (*Prevotella* OTU, Spearman rho = 0.68; *p* < 0.0001), the NP (*Mitis Group Streptococcus* Spearman rho = 0.39; *p* = 0.001) or both upper airway specimens (*H. influenzae*, Spearman rho = 0.5, *p* < 0.0001), suggesting similarity of lavage microbiota to those in both the OP and NP.

### Lavage microbiota were distinct from OP and NP microbiota

PERMANOVA detected significant differences between the microbiota in both the OP (*p* = 0.0001, Pseudo-*F* 12.2, *df* 1) and the NP (*p* = 0.0001; Pseudo-*F* 21.9; *df* 1) when compared to Lavage-1; however, this difference was more pronounced between NP and Lavage-1 specimens, as indicated by a higher Pseudo-*F* value (Table 3). Bray-Curtis similarity between paired OP and Lavage-1 specimens was >50 % for 37/65 (57.8 %) children, compared with only 5/65 (7.7 %) children when NP and Lavage-1 microbiota were compared. Two children had >50 % Bray-Curtis similarity between the Lavage-1 microbiota and that of the paired OP and NP swabs, whereas for 25/65 (38 %) children, the Lavage-1 microbiota were <50 % similar to that in either of the paired OP or NP swabs.

### Combining OP and NP data increased overall similarity with the lavage microbiota, but significant differences remained

We next combined the OP and NP data to determine whether an upper airway sampling strategy that included both sites concurrently would provide a more comprehensive representation of Lavage-1 microbiota. The combined OP and NP data were significantly different to Lavage-1 (PERMANOVA *p* = 0.0001; Pseudo-*F* 6.8, *df* 1); however, the Pseudo-*F* statistic was 1.8 and 3.2 times lower than comparisons of Lavage-1 with the OP or NP microbiota, respectively (all *df* 1; Table 3). Furthermore, the PERMANOVA components of variation showed that differences in the microbiota among specimen types contributed 25.3, 17.9 and 13.0 % of variation between NP, OP and combined upper airway data, respectively, when compared to Lavage-1. These findings indicate that the combined OP and NP data provided a better representation of Lavage-1 microbiota than analysis of either upper airway site alone; however, significant differences remained.

We then tested whether combined Lavage-1 and Lavage-2 data would show higher similarity to the upper airway microbiota than analysis of Lavage-1 alone. This analysis was performed for the subset of 49 children with paired Lavage-2 data. Inclusion of Lavage-2 data increased the richness detected from the lower airways by 2–27 OTUs (median 8, 95 % CI 6–12) for 27/49 children. Overall, Bray-Curtis similarity between the combined OP and NP data (upper airway microbiota) and the combined Lavage-1 and Lavage-2 data (lower airway microbiota) was >50 % for 34/49 (69.4 %) children. A PERMANOVA test showed higher similarity between the upper and lower airway data after inclusion of Lavage-2 (Pseudo-*F* 4.9, *df* 1; Table 3); however, significant differences remained (PERMANOVA *p* = 0.0001) with 12.1 % of the total variation explained by variation among the upper and lower airway microbiota. Variation in the microbiota due to differences between the children explained 45.1 % of the total variance (*p* = 0.0001; Pseudo-*F* 3.2, *df* 48), while 42.8 % of the data variability remained unexplained.

**Fig. 5:**
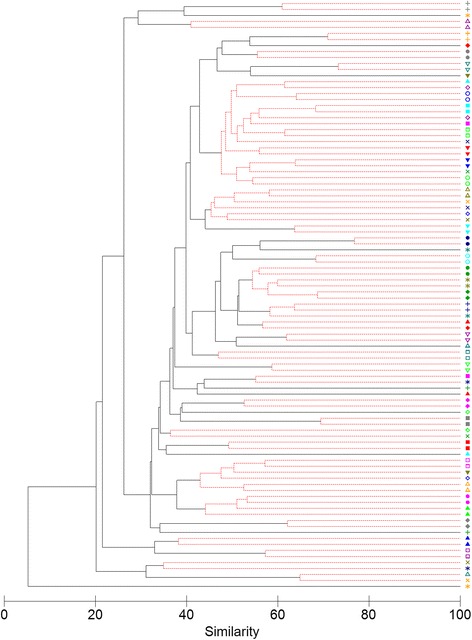
Paired specimens from 65 % of children were more similar to each other than to specimens from other children. Hierarchical group average cluster analysis based on Bray-Curtis similarity matrix derived from square root transformed OTU-level data. Forty-nine children with sequence data for upper airway (combined NP and OP) and lower airway (combined Lavage-1 and Lavage-2) microbiota were included in the cluster analysis. *Coloured symbols* at the right of the figure indicate paired upper and lower airway data from individual children. A similarity profile permutation test was used to identify clusters with significant similarity (*red dashed branches*)

Significant differences detected in the PERMANOVA test likely reflect a subset of children with low similarity observed between the lower and upper airway data. This was evident in hierarchical group average cluster analysis that showed paired upper and lower airway data from 33/49 (67.3 %) children clustered together with significant similarity, whereas no significant similarity was detected for 16/49 (32.7 %) children (Fig. 5). As a comparison, paired OP and Lavage-1 specimens from only 27/65 (41.5 %) children clustered together in hierarchical cluster analysis (Additional file 1: Figure S4). Clustering of paired upper and lower airway data was not associated with recent antibiotic use or low bacterial load in any of the specimens (all comparisons Mann-Whitney *U* test *p* > 0.05); 7/16 (44 %) children with low similarity between paired upper and lower airway data and 16/33 (49 %) children with high similarity had high bacterial load in all specimens. Low similarity between the upper and lower airway microbiota was also unrelated to the diagnostic group; the lower and upper airway microbiota from 11/31 (35 %) children with CSLD, 4/13 (31 %) children with PBB and 1/5 (20 %) control children failed to cluster together. Collectively, these data indicate that the highest similarity between the microbiota in the different airway sites was obtained when the upper (combined NP and OP swabs) and lower airway (combined Lavage-1 and Lavage-2) data were compared; however, significant overall differences remained due to low similarity between paired data from a subset of children.

### Microbiota in lower and upper airways discriminated clinically defined groups

As paired upper and lower airway data from 67.3 % children clustered together despite the overall significant difference in the microbiota, we next tested whether discrimination of disease groups based on analysis of lower airway microbiota would have been detected if only the upper airway data were analysed. This was done by testing for differences in the lower airway microbiota related to each disease group and then determining if any observed discrimination was reproduced in the analysis of the upper airway microbiota.

**Fig. 6:**
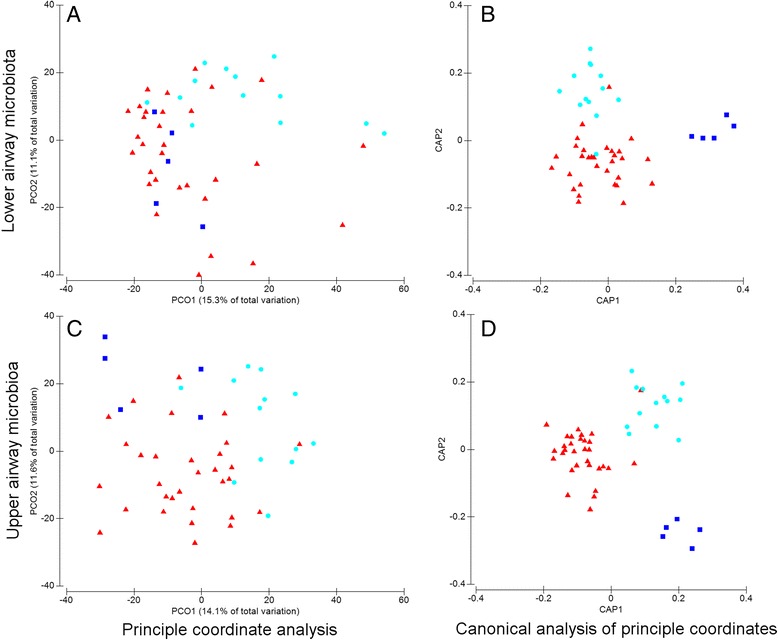
Significant discrimination of microbiota in different diagnostic groups detected for both the lower and upper airway data. Differences in the microbiota from children with CSLD (*red triangles*) and PBB (*light blue circles*) and control children (*dark blue squares*) were evident in principal coordinate analysis of the lower (**a**) and upper (**c**) airway data. In the lower airway plots, each data point represents the combined Lavage-1 and Lavage-2 data from a single child. Likewise, in the upper airway plots, each data point represents the combined NP and OP data from a single child. Canonical analysis of principal coordinates (CAP) from the lower (**b**) and upper (**d**) airway data discriminated the diagnostic groups. Each CAP included 49 children. CAP eigenvalues were all >0.8. The misclassification error was 26.5 and 12.2 % for the lower and upper airway data, respectively. CAP of lower and upper airway specimens were optimised with 26/49 and 18/49 PCO axes, respectively

PERMANOVA detected significant differences in the lower airway microbiota among the disease groups (*p* = 0.0001; Pseudo-*F* 2.46; *df* 2), with 29.2 % of the total variation in the microbiota explained by differences among diagnostic groups. Clustering of data points from each diagnostic group was suggested in the PCoA (Fig. 6a); however, there was an overlap between the groups with only 26.4 % of the total variation explained by PCO1 and PCO2. Canonical analysis of principal coordinates (CAP) was then used to determine if other axes would better discriminate the microbiota based on the a priori defined diagnostic groups. The CAP analysis detected significant differences between the lower airway microbiota based on the diagnostic group (eigenvalues CAP1 0.86, CAP2 0.80; misclassification rate 26.5 %; trace statistic *p* = 0.02) with distinct separation of the groups across the CAP axes (Fig. 6b).

This group-specific discrimination was reproduced in the analysis of the upper airway microbiota (PERMANOVA *p* = 0.0001; Pseudo-*F* 3.59, *df* 2). Dispersion of the upper airway data in PCoA was different to that of the lower airways (Figs. 6a, c), reflecting underlying differences in the diversity between the two anatomic sites. As with the lower airway data, there was some overlap between data points for each diagnostic group in the PCoA that was resolved by CAP analysis (eigenvalues CAP1 0.89, CAP2 0.85; misclassification rate 12.2 %; trace statistic *p* = 0.0001) with distinct separation of the groups across the CAP axes (Fig. 6d). These data indicate that differences between each diagnostic group were detected regardless of whether the lower or upper airway microbiota data were analysed.

SIMPER analysis was then performed to identify OTUs that contributed most to the significant dissimilarity in the microbiota between the diagnostic groups. SIMPER analyses were limited to comparisons of children with CSLD or PBB, as paired upper and lower airway data were available for only five control children (too few to allow robust analysis). For SIMPER analysis comparing the microbiota in children with CSLD or PBB, the top 10 OTUs contributed 30 and 26 % of the variation in the lower and upper airway data, respectively. Seven of the 10 OTUs contributing most to discrimination of the diagnostic groups based on analysis of lower airway data also discriminated the groups in the analysis of the upper airway data (Table 4). Furthermore, the top four OTUs contributing to discrimination of the diagnostic groups (consistent with *Moraxella catarrhalis*, *H. influenzae*, *Prevotella* sp. and Mitis Group Streptococci) were the same in analysis of the lower and upper airway data (Table 4); these four OTUs collectively explained 17.8 % the variation in the lower airway microbiota and 14.9 % of the variation in the upper airway data between the diagnostic groups.

**Table 4:** Results of SIMPER analysis comparing the microbiota in children with PBB and CSLD

Site	OTU	BLASTn (% identity)
Lower airway data	Otu00003	*Moraxella catarrhalis* (98)
	Otu00002	*Haemophilus influenzae* (99)
	Otu00004	*Prevotella* sp. (98)
	Otu00001	Mitis Group Streptococci (99)
	Otu00011	*Neisseria lactamica* (99)
	Otu00010	*Neisseria* sp. (99)
	Otu00005	*Haemophilus haemolyticus* (99)
	Otu00006	*Porphyromonas* sp. (99)
	Otu00009	*Prevotella melaninogenica* (100)
	Otu00030	*Leptotrichia* sp.(88)*
Upper Airway Data	Otu00003	*Moraxella catarrhalis* (98)
	Otu00002	*Haemophilus influenzae* (99)
	Otu00001	Mitis Group Streptococci (99)
	Otu00004	*Prevotella* sp. (98)
	Otu000012	*Corynebacterium propinquum* (100)
	Otu00005	*Haemophilus haemolyticus* (99)
	Otu00008	*Staphylococcus aureus* (100)
	Otu00006	*Porphyromonas* sp. (99)
	Otu000010	*Neisseria* sp. (99)
	Otu000017	*Moraxella lincolnii* (98)

SIMPER analysis was performed to determine the OTUs that contributed most to the observed significant dissimilarity in the microbiota between diagnostic groups. Results are shown for the 10 OTUs in the lower and upper airway data that contributed most to the differences between children with CSLD and PBB. In comparison of children with CSLD or PBB, these OTUs contributed 30 and 26 % of the observed variation in the lower and upper airway microbiota, respectively. Comparisons with control children were not done as too few control children (*n* = 5) had paired upper and lower airway data to allow robust assessment of explanatory variables. OTUs are listed in a decreasing order of variation explained. BLASTn identities were selected based on 100 % coverage and >98 % identity score (% identity). OTU00030 was identified by BLASTn as Leptotrichia (*), but with only 88 % identity

**Fig. 7:**
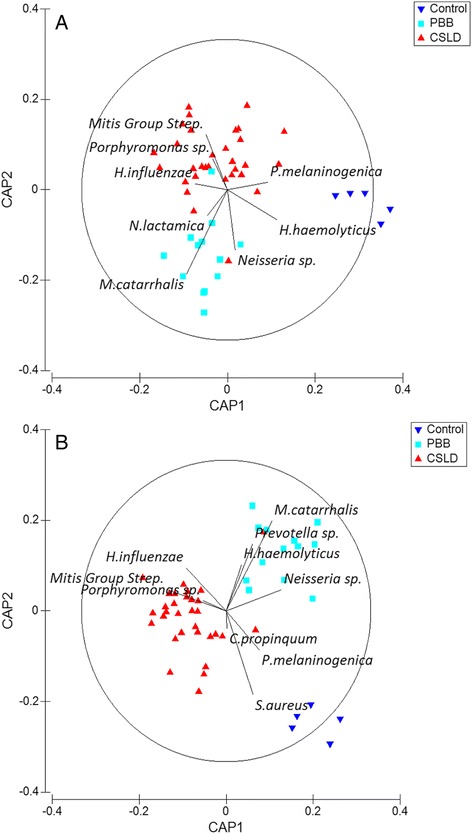
Analysis of OTUs contributing to discrimination of clinically defined groups based on analysis of the lower or upper airway microbiota. SIMPER analysis was used to identify the top 10 OTUs contributing to discrimination of the CSLD and PBB groups. These OTUs were visualised on the CAP ordination with vectors whose length and direction reflected the Spearman rank correlations of these OTUs with the CAP axes. For the lower airway data (**a**), OTU vectors consistent with a *Prevotella* sp. and a *Leptotrichia* sp. are not shown as they overlapped with the *M. catarrhalis* and the *Neisseria* sp. vectors, respectively. For the upper airway data (**b**), the OTU vector consistent with *M. lincolnii* is not shown as it overlapped with the *Porphyromonas* sp. vector

We then visualised relationships between OTUs identified by SIMPER analysis and the CAP axes. This was done using a Spearman rank correlation between each OTU and the CAP axes [27] with results represented as a vector plot (Fig. 7). For the lower airway data (Fig. 7a), the CAP2 axis best discriminated the CSLD and PBB groups. Dispersion across CAP2 was associated with OTUs consistent with Mitis Group Streptococci, *H. influenzae*, a *Porphyromonas* sp. and *Prevotella melaninogenica* (discriminating the CSLD group), and *M. catarrhalis*, a *Neisseria* sp., a second *Prevotella* sp. and *Haemophilus haemolyticus* (discriminating the PBB group).

Similar findings were observed in the analysis of the upper airway data, except that the CSLD and PBB groups were discriminated across the CAP1 axis (Fig. 7b). As with the lower airway data, discrimination of the groups was associated with OTUs consistent with Mitis Group Streptococci, *H. influenzae*, a *Porphyromonas* sp. and *Moraxella lincolnii* (discriminating the CSLD group), and *M. catarrhalis*, a *Neisseri*a sp., 2 *Prevotella* sp., *H. haemolyticus* and *Staphylococcus aureus* (discriminating the PBB group). The OTU consistent with *C. propinquum* contributed to separation across CAP2, but not CAP1 (Fig. 7b). Collectively, these data show that the observed discrimination of the CSLD and PBB groups was associated with similar OTUs in analysis of both the lower and upper airway data.

## Discussion

We have shown that the combined OP and NP microbiota provided a better representation of BAL microbiota for most children in our study (67.3 %) than the analysis of the microbiota in either the upper airway site alone; however, low similarity between the paired upper and lower airway microbiota was detected for 32.7 % of the children. Despite low similarity in some children, a CAP analysis showed that a non-invasive sampling strategy that combined OP and NP swabs discriminated clinically defined groups of children as well as the microbiota in invasive lavage specimens. This observation was supported by results of a SIMPER analysis that showed seven of the top 10 OTUs contributing to discrimination of the lower airway data from children with CSLD and PBB and also contributed to discrimination of clinical groups in analysis of the upper airway data. Collectively, these results suggest that combined NP and OP sampling provided an imperfect, but reliable, measure of airway microbiota for most children in this study. Based on these results, we recommend that both OP and NP specimens are included when measures of upper airway microbiota are used to study lower airway disease in young children who do not expectorate.

Our results showing combined OP and NP provided the best representation of BAL microbiota likely reflects the importance of microbes in the nasopharynx to the lower airway infection in young children. Studies of adults and older children have reported high similarity between the OP and the lower airway microbiota and concluded that the lung microbiota develops from microaspiration of oral flora [1, 13]. Seeding of the lower airways by NP microbiota during episodes of rhinorrhoea has been suggested [28], but there have been limited data to date to support this hypothesis. Episodes of rhinorrhoea associated with upper respiratory tract infection are common in young children [29, 30]. Furthermore, the nasopharynx is an important reservoir of respiratory pathogens in paediatric populations [14, 16, 31], with NP pathogen carriage associated with the increased risk of lower airway infection [32]. Several studies have reported associations between NP microbiota and lower airway diseases including pneumonia [17], bronchiolitis [33] and asthma [18]. A longitudinal study of NP aspirates from 234 children under 1 year of age identified the NP microbiota as a determinant for infection spreading to the lower airways [18]. Culture-based studies have also shown high concordance between the presence of pathogenic species and strains in the nasopharynx and BAL of young children with bronchiectasis [16]. While these findings support a role for the NP microbiota seeding the lower airways of young children, it is unclear if this only occurs during episodes of rhinorrhoea or is related to other mechanisms. Our data highlight the need for further research to understand mechanisms of seeding the lower airways by NP microbiota, particularly in paediatric populations.

The low similarity between the paired upper and lower airway data in 32.7 % of the children was unrelated to recent antibiotic treatment, low bacterial load or diagnostic group. Earlier studies also detected subsets of individuals with dissimilarity between the upper and lower airway specimens. Bassis et al. [1] compared oral wash and BAL microbiota in 28 healthy adults and found that although the microbiota in the different specimen types showed significant overlap, the microbiota in paired specimens from 57 % of the study cohort were dissimilar. Likewise, Zemanick et al. [8] reported overlap between the microbiota in OP swabs and sputum specimens from older children and young adults with cystic fibrosis, with significant divergence between oral and sputum microbiota in a subset of the cohort related to increased relative abundance of potentially pathogenic genera in sputum specimens. The reasons for the dissimilarity between the paired upper and lower airway specimens in our study are unclear but may indicate either a role for other upper airway bacterial reservoirs in seeding the lower airways (for example, the sinuses [4, 34, 35]) or lower airway environmental pressures that select for specific microbiota [1, 8].

Our results highlight the importance of considering the generalizability of airway microbiota data. Our observations are specific to a cohort of young children (median age 2.2 years) with PBB, CSLD or no lung disease. In contrast, findings from other studies have been specific to populations of healthy adults [1]; older children, adolescents and adults with cystic fibrosis [8, 10, 13]; and patients with end-stage respiratory disease [9, 11]. At the extremes of this spectrum, studies of healthy adults have demonstrated correlation between the OP and the lower airway microbiota [1], whereas studies of end-stage respiratory disease have demonstrated divergence of the microbiota from the lung and OP specimens [9, 11]. Our finding that lavage microbiota showed similarity to both OP and NP microbiota may not be generalizable to other paediatric or adult populations or to children with end-stage respiratory disease; however, this limitation is common to all studies of respiratory microbiota in defined populations. Understanding the similarity and divergence of the microbiota across the airways in populations of different age and clinical presentation remains an important area for future research.

The clinical implications of our findings are unclear. The CAP analysis suggests lower and/or combined OP and NP microbiota profiles may differentiate CSLD and PBB. The OTUs discriminating the CSLD and PBB groups were consistent with the taxa previously reported in the NP of young Dutch children [14] and in the NP, OP and BAL of healthy adults [1]. Biesbroek et al. [14] reported fewer respiratory infections in children with NP colonisation by *Moraxella*, *Corynebacterium* and *Dolosigranulum* species compared to children colonised by *Streptococcus* and *Haemophilus* species. In our study, OTUs consistent with Mitis Group Streptococci and *H. influenzae* were associated with children with CSLD, whereas *M. catarrhalis* was associated with PBB. Bassis et al. [1] reported enrichment of *Prevotella* sp. in the lung, whereas in our study, *Prevotella* in the upper airway data was associated with the PBB group.

Our results must, however, be considered within the limitations of our study design (a cross-sectional analysis with imbalances between the clinically defined groups). Although the study design was suitable for assessing the similarity of the lower and upper airway microbiota, potential confounding factors limit clinical inference. For example, 83 % of the children with CSLD were Indigenous Australians compared with only 7 % of children with PBB and none of the controls. No studies have compared the upper or lower airway microbiota in Indigenous and non-Indigenous children; however, culture and PCR-based studies have reported significant differences in the multiplicity and density of potential respiratory pathogens colonising the nasopharynx of Indigenous compared to non-Indigenous Australian children [36, 37]. Furthermore, an earlier study comparing the microbiota in non-Indigenous Australian children with PBB and CSLD did not detect disease-associated microbiota and instead reported a core BAL microbiota common to both disease groups [5]. Thus, it is unclear whether the discrimination observed in our study indicates disease-specific microbiota profiles or underlying population-level differences. Likewise, we cannot exclude potential confounding due to antibiotic treatment, as 65 % of the children with CSLD had recently received antibiotics compared with ~20 % of controls and children with PBB. Further investigation to confirm whether upper and/or lower airway microbiota profiles differentiate CSLD and PBB is warranted but was beyond the scope of the current study. Further study is also needed to understand whether airway microbiota in Indigenous Australian children is different to that of non-Indigenous children and whether such differences may relate to the disproportionately high rates of CSLD in Indigenous children [38].

Age-related factors may also confound analysis of disease-specific differences in airway microbiota. Longitudinal studies have reported age-related changes in NP microbiota [14], whereas we did not detect associations between age and bacterial load or diversity in any of the specimen types. Cross-sectional analysis of age-related changes in the airway microbiota of children with chronic lung diseases may also be confounded by differences in the age at disease-onset, duration of disease, and disease severity. Such data were not available for inclusion in our analysis.

Consistent with earlier reports [17, 39, 40], a high proportion (31 %) of DNA extracts in our study had bacterial loads below that recommended for 16S rRNA gene sequencing [24, 25]. A significant role for contaminant OTUs in sequence data from low bacterial load specimens was recently recognised [24]. The method we used to exclude contaminant OTUs was reported previously for analysis of low bacterial load upper airway and middle ear specimens from otitis media patients [26]. While this method proved useful, allowing successful characterisation of the microbiota in 77/94 specimens with low to very low bacterial load, it remains possible that further refinement of the current methods may improve detection and eradication of contaminant OTUs. Refinement of such algorithms was beyond the scope of the current study but should remain an area of active research.

Low bacterial load in our specimens is unlikely to reflect a sampling bias, as all specimens were collected using the same standardised protocol while children were under general anaesthesia. Furthermore, low bacterial load was consistently detected across the NP and lavage specimens from 15/78 children in our study, suggesting it may be a feature of the airways in specific children. The clinical significance of this observation is unclear; however, low bacterial load in airway specimens has been associated with better clinical outcomes in patients with respiratory infections [6, 41]. Alternative extraction methods may also increase bacterial DNA yield from low biomass specimens [25] and, thus, improve microbiomic characterisation of paediatric airway specimens [42]. This may be especially important if NP swabs are included in upper airway sampling strategies.

Several technical limitations should also be considered when interpreting results from this study. All specimens were placed in skim milk tryptone glucose glycerol broth (STGGB )media prior to long-term storage at −80 °C. STGGB is a preservative media optimised for recovery of respiratory pathogens [43] and is widely used in NP carriage studies [44]. While it is possible that storage in STGGB may have permitted overgrowth of some taxa, we believe this is unlikely as the specimens were kept on ice after collection and were stored at −80 °C within 2 h of collection. We also cannot exclude potential contamination from residual bacterial DNA in the bronchoscopes as instrument background controls were not collected.

## Conclusions

We have shown that for most children in our study, an upper airway sampling strategy that combined OP and NP microbiota provided better representation of BAL microbiota than analysis of either upper airway site alone. Our data suggest that the lower and upper airway microbiota profiles may differentiate children with CSLD or PBB; however, further testing is needed to confirm this observation as our study was not optimally designed for clinical inference. We conclude that although upper airway sampling provided an imperfect representation of the lower airway microbiota, it could be used as a reliable measure of airway microbiota for most children in this study. Sampling strategies that include both OP and NP specimens are recommended when non-invasive upper airway sampling is needed to assess airway microbiota in young children who do not expectorate.

## Methods

This study was approved by the Human Research Ethics Committee (HREC) of the Northern Territory Department of Health and Menzies School of Health Research, Darwin (HREC 07/63 and 09/02), and the Royal Children’s Hospital, Brisbane (HREC 2003/017 and 200800064). Written informed consent was obtained from the parent/carer of each child.

### Clinical definitions

PBB was clinically defined based on the presence of a wet cough for >4 weeks, the absence of specific causal indicators for cough (e.g., failure to thrive, chest wall deformity) and resolution of cough within 2 weeks of commencing appropriate antibiotic therapy [45]. CSLD was clinically defined based on the presence of a wet productive cough for >8 weeks, with a clinical profile of bronchiectasis without cHRCT findings (either because cHRCT was not undertaken or did not fulfil radiological criteria) [46]. Bronchiectasis diagnosed by cHRCT was based on an inside bronchial diameter-to-adjacent artery ratio >0.8 [47]. None of the children with bronchiectasis had cystic fibrosis.

### Study cohort and specimens

The study cohort was a subset of children <10 years of age sequentially enrolled in either (i) an observational study of paediatric CSLD at Royal Darwin Hospital, Darwin, Australia, between October 2011–October 2012 or (ii) an observational study of children undergoing bronchoscopy at the Royal Children’s Hospital, Brisbane, Australia, between February 2011 and November 2012 [48, 49]. Children (<10 years of age) undergoing flexible bronchoscopy for a clinical indication, excluding cystic fibrosis, were eligible for inclusion in the Brisbane-based study [49]. Children undergoing cHRCT and flexible bronchoscopy for investigation of chronic cough and suspected bronchiectasis were eligible for the Darwin-based study [48]. The CSLD cohort included all children prospectively recruited to the Darwin-based study and five randomly selected children from the Brisbane-based study. The PBB and control cohorts included all children with these diagnoses prospectively recruited to the Brisbane-based study who had sufficient specimens stored for bacterial analyses (28/30 children with PBB and 10/12 disease controls). The control children had undergone bronchoscopy for assessment of other clinical presentations (e.g. stridor, recurrent croup, unexplained dyspnea) associated with underlying diagnoses of tracheomalacia (*n* = 6), laryngomalacia, and bronchomalacia (*n* = 2), granulation on the vocal cords (*n* = 1) and an absent cardiac segment (*n* = 1).

An OP swab, NP swab and two sequential BAL specimens were collected while the children were under general anaesthesia. Identical specimen collection protocols were used for the Darwin- and Brisbane-based studies [48, 49]. The OP and NP swabs were collected immediately prior to insertion of the bronchoscope. The NP swabs were collected by placing a rayon-tipped swab into the nasopharynx via a single nostril; the swab was rotated for 2–3 seconds before being withdrawn and placed in a tube containing 1 mL STGGB. The OP swabs were collected by swabbing the anterior and posterior pillars then placed in 1 mL of STGGB. Bronchoscopy with collection of BAL was performed as per the European Respiratory Society guidelines [50]. The BAL were collected using two volumes of sterile normal saline (1 mL/kg to a maximum of 10 mL for the first volume, Lavage-1; and 2 mL/kg to a maximum of 20 mL for the second volume, Lavage-2) which was instilled into the lobe and suctioned immediately into a mucus trap. Lavage-1 sampled the most affected lobe (as determined by cHRCT) or the right middle lobe if cHRCT results were not available. Lavage-2 was collected from the same lobe as Lavage-1. The aliquots of Lavage-l and Lavage-2 were diluted in equivalent volumes of 2× concentrated STGGB for bacterial analyses. All specimens were transported to the laboratory on ice bricks for processing and storage within 2 hours, with all specimens in STGGB stored at −80 °C prior to bacterial analyses.

### Bacterial analyses

Bacterial analyses were performed using DNA extracted from 200 μL of swab media (OP and NP swabs) and 400 μL of BAL in STGGB (equivalent to 200 μL of neat BAL). DNA extraction was done using QIAamp columns (QIAgen) with bead beating pre-treatment as detailed in Additional file 1. A dedicated DNA aliquot was used to determine the total bacterial load in each extract. The bacterial load was estimated using qPCR targeting the 16S rRNA gene [23], as described previously [22]. Briefly, each 10 μL qPCR reaction included 1× SensiMix™ SYBR® reagent (Bioline), 300 nM of each primer (forward primer: 5′- TCCTACGGGAGGCAGCAGT -3′; reverse primer: 5′- GGACTACCAGGGTATCTAATCCTGTT -3′) and 1 μL of template DNA. The reaction conditions were an initial hold at 50 °C for 2 min followed by incubation at 95 °C for 10 min then 35 cycles of 95 °C for 15 s, 58 °C for 15 s and 72 °C for 45 s. Melt-curve analysis was then done between 80 and 90 °C with 0.1 °C steps. Low bacterial load was defined as <10^3^ GE/μL extracted DNA, as this level was recommended previously for 16S rRNA gene sequencing analyses [24, 25].

Sequencing of the 16S rRNA gene V1-3 regions was performed using a Roche 454 GS FLX Titanium platform as detailed in Additional file 1. Four DNA extraction negative controls and two aliquots of a reproducibility control were also sequenced. The reproducibility control was used to measure variation between sequencing batches. It contained DNA extracted from a pool of five OP swabs that were all taken from an adult volunteer. DNA was extracted from the pooled OP swabs then aliquoted and stored at −80 °C prior to sequencing. Results for the negative and reproducibility controls are detailed in Additional file 1. Raw sequence data are available upon request and subject to permission from the relevant Human Research Ethics Committees.

Quality filtering of sequence data was done using mothur (version 1.33.3) [51] according to the 454-SOP [52, 53] with the following modifications: the sff.multiple command was used to combine data from multiple sff files; sequences with one or more mismatches in the barcode were excluded. OTUs were defined based on 97 % sequence similarity and taxonomic classifications were assigned using the SILVA reference alignment (release 102) [54]. Where reference to the SILVA alignment did not identify OTUs beyond the family level, BLASTn [55] was used to assign taxonomy based on 100 % coverage and >98 % identity. Additional filtering was performed to exclude probable contaminant OTUs and sequencing errors (referred to collectively as probable contaminant OTUs) that arise during amplification of specimens with low bacterial load. This was done as earlier studies have demonstrated an inverse relationship between bacterial load and detection of spurious OTUs [24, 26]. Probable contaminant OTUs arising from sequencing of specimens with low bacterial biomass and contaminants detected in negative controls were identified and excluded from further analysis as described in Additional file 1. A complete list of all OTUs excluded from downstream analyses is presented in Additional file 1: Table S1. Following exclusion of contaminant OTUs, subsampling to a depth of 1025 reads was performed based on review of rarefaction curves and Good’s coverage. Specimens with fewer than 1025 reads were excluded from further analysis [24, 26].

### Statistical analyses

Biostatistical analyses were performed using Stata/IC version 14.0 (StataCorp LP, USA). All tests were two-tailed; significant results were indicated by a *p* value of <0.05 unless otherwise stated. Median with binomial-based 95 % CI was used to describe data that were not normally distributed. The non-parametric Mann-Whitney *U* test was used to test for significant differences between two groups. Comparison of multiple groups was done using a non-parametric Kruskal-Wallis test followed by a post hoc Dunn’s test with Bonferroni correction for pairwise comparisons. Statistical dependence between continuous variables was determined using a Spearman’s rank correlation. Box and whisker plots were generated to show the interquartile range (IQR; boxes), median (line within the box), upper and lower adjacent values (whiskers) and outliers (dots). Graphical figures were prepared with Stata/IC or R version 3.2.3 [56] with the ggplot2 [57] and beeswarm [58] packages.

Analyses of alpha and beta diversity in the microbiota were performed using PRIMER (version 6.1.15) [27, 59]. Simpson’s index of diversity (1-D) was used to assess the diversity of the bacterial community structures; this measure takes OTU richness and relative abundance into account. Beta diversity was assessed using a Bray-Curtis similarity matrix based on square root transformed OTU-level data. The SIMPER routine was used to identify OTUs contributing most to variation between specimens and diagnostic groups. Hierarchical group average cluster analysis was performed with similarity profile permutation test to determine if paired specimens clustered together with significant similarity. Similarity between the microbiota in different specimens was visualised using unconstrained principal coordinate analysis. PERMANOVA was used to test for significant differences between the microbiota in different specimen types. The PERMANOVA was done using a crossed-design with specimen type designated as a fixed factor and child designated as a random factor. The Type III sums of squares method was used with permutation of residuals under a reduced model and 9999 permitted permutations. One-way PERMANOVA to test for significant differences between the microbiota in each diagnostic group was performed using the Type III sums of squares method with unrestricted permutation of raw data. Where PERMANOVA detected significant differences in the microbiota between clinically defined groups, a discriminant canonical analysis of principal coordinates (CAP) was used to identify principal coordinates that best explained variation in the microbiota. In contrast to principal coordinate analysis which identifies axes that best explain the overall variability in the data, CAP is a constrained test that identifies principal coordinates that best discriminate a priori defined groups [27]. The CAP analysis was done to identify the principal coordinates with the lowest misclassification error (equivalent to those that best discriminated the groups). CAP diagnostics were performed using a leave-one-out allocation procedure to identify the number of axes (*m*) required to minimise the misclassification error. The CAP eigenvalues of the squared canonical correlations were used to indicate the ability of the corresponding axes to separate the a priori defined groups. The significance of differences detected by CAP analysis was determined using the permutation-based trace statistic [27].

## Abbreviations

BAL, bronchoalveolar lavage; CAP, canonical analysis of principal coordinates; cHRCT, chest high-resolution computed tomography; CSLD, chronic suppurative lung disease; *df*, degrees of freedom; GE, genome equivalents; NP, nasopharyngeal; OP, oropharyngeal; OTUs, operational taxonomic units; PBB, protracted bacterial bronchitis; PCO1, first principal coordinate axis; PCO2, second principal coordinate axis; PCoA, principal coordinates analysis; PERMANOVA, permutational multivariate analysis of variance; SIMPER, similarity percentages routine

## Additional file

Additional file 1: Supplementary materials. **Figure S1:** Relative abundance of contaminant OTUs in different specimen types. **Figure S2**: Principal coordinate analysis demonstrating that specimens with low bacterial load did not cluster discretely from those with >10^3^ GE/μL extracted DNA. **Figure S3**: Bacterial diversity in each specimen type separated by diagnostic group. **Figure S4**: Similarity of OP swab and Lavage-1 microbiota. **Figure S5**: Scatterplots representative of relationships observed for potential contaminant taxa in clinical specimens. **Table S1**: Probable contaminant OTUs removed prior to downstream analyses. (DOCX 939 kb)
