# Supplementary material for: The microbiota in bronchoalveolar lavage from young children with chronic lung disease includes taxa present in both the oropharynx and nasopharynx
Source: Microbiome. 2016 Jul 7;4:37. doi: 10.1186/s40168-016-0182-1 (PMC4936249; doi:10.1186/s40168-016-0182-1)
Supplement: Supplementary file 1 — Supplementary materials. Figure S1: Relative abundance of contaminant OTUs in different specimen types. Figure S2: Principal coordinate analysis demonstrating that specimens with low bacterial load did not cluster discretely from those with >103 GE/μL extracted DNA. Figure S3: Bacterial diversity in each specimen type separated by diagnostic group. Figure S4: Similarity of OP swab and Lavage-1 microbiota. Figure S5: Scatterplots representative of relationships observed for potential contaminant taxa in clinical specimens. Table S1: Probable contaminant OTUs removed prior to downstream analyses. (DOCX 939 kb) [file 40168_2016_182_MOESM1_ESM.docx]

**Additional File 1**

***DNA extraction***

DNA was extracted from 200 µL of swab media (OP and NP swabs) and 400 µL of BAL in STGGB (equivalent to 200 µL of neat BAL). Specimens were thawed on ice then vortexed on high speed for 15 s. Aliquots of each specimen were taken to a new tube and cellular material was pelleted by centrifugation. The pellet was resuspended with 600 µL of Buffer AL (QIAgen) and the suspension applied to 0.69 g of 0.1 mm silica spheres in 2 mL tubes (MP Biomedicals Lysing matrix B) for bead-beating in a FastPrep (MP Biomedicals) at 6.0 m/s for 30 s. Following bead-beating, the supernatant was removed and 200 µL of 100% ethanol was added. The mixture was vortexed then DNA was extracted using QIAamp columns as per the manufacturer’s instructions (QIAamp DNA Mini and Blood Mini Handbook, 04/2010). DNA was eluted with 60 µL QIAgen buffer AE. Four reagent only DNA extraction negative controls were included.

***16S rRNA gene sequencing***

Bacterial tag encoded FLX amplicon pyrosequencing (bTEFAP) [[1](#_ENREF_1)] of the 16S rRNA gene was performed by a commercial provider (Molecular Research DNA, USA). The V1-3 region was amplified using primers 27Fmod (5’ AGRGTTTGATCMTGGCTCAG 3’) and 519modbio (5’ GTNTTACNGCGGCKGCTG 3’). PCR amplification was done using HotStarTaq Plus Master Mix (Qiagen, USA) with the following cycling conditions: 94°C for 30s; 28 cycles of 94°C for 30s, 53°C for 40s and 72°C for 1min; and 72°C for 5mins. PCR amplicons from different specimens were mixed at equimolar concentrations and purified using Agencourt Ampure beads (Agencourt Bioscience Corporation). Sequencing was performed on a Roche 454 GS FLX Titanium platform as per the manufacturer’s guidelines. Sequencing was performed in two batches with specimens from individual children tested together in the same batch. There was no significant difference between the microbiota data from the first and second sequencing batches (ANOSIM p=0.528; R= -0.004). Replicates of the reproducibility control between sequencing batches had Bray-Curtis similarity of 71% and 90% at the OTU- and family-levels, respectively.

***OTUs detected in DNA extraction negative controls***

Two DNA extraction negative controls were included with each sequencing batch. No bacteria were detected in these controls when tested by the total bacterial load qPCR and no sequences were returned for negative controls from the first sequencing batch.

Negative controls tested with the second sequencing batch returned 458 and 1119 reads, respectively. These reads represented 24 OTUs with only 7 OTUs (29%) common to both negative controls. These OTUs were not systemically present in data from the clinical specimens, with 66/85 specimens from this sequencing batch sharing <2 OTUs with the negative controls, and 13/85 sharing none. In the 19 specimens, only 3-8 OTUs were shared with the negative controls (median 4; 95% CI 4-5). Collectively, these data are inconsistent with detection of systemic contamination.

As contaminant OTUs are expected in low bacterial biomass specimens[[2](#_ENREF_2)], the OTUs detected in the negative controls were examined to determine if their relative abundance was inversely correlated with the total bacterial load in the DNA extract[[3](#_ENREF_3)]. Overall, 23/24 OTUs detected in at least one negative control were either i) only present in a negative control (i.e. not detected in any clinical specimen); ii) showed an inverse correlation (Spearman rho between -0.7 and -1.0) between their relative abundance and the total bacterial load in the DNA extract when detected in clinical specimens; or iii) were only detected at relative abundance >1% in samples with bacterial load <10^3^ GE/µL extracted DNA. Thus, these OTUs are all consistent with detection of contaminant OTUs as a result of the low bacterial biomass in the negative controls, and were excluded from downstream analyses (Additional Table 1).

OTU00003 (a *Moraxella* sp.) was detected at <0.5% relative abundance in one of the two negative controls from the second sequencing batch. This OTU did not show an inverse correlation with the total bacterial load in DNA extracts and, thus, is inconsistent with detection of a contaminant sequence in specimens with low bacterial biomass (Additional Figure 5). This OTU was also detected in 56/201 clinical samples tested in the first sequencing batch, but not the corresponding negative controls. As these data do not support detection of this OTU as a contaminant, OTU00003 was not excluded from downstream analyses.

***Probable contaminant OTUs in low biomass clinical samples***

The data from clinical specimens were reviewed to determine if taxa reported previously as reagent and kit contaminants[[2](#_ENREF_2)] were present. Overall, 46/93 genera reported previously as potential contaminants[[2](#_ENREF_2)] were present in the data. Where potential contaminant genera were present in >20 specimens, a Spearman rank correlation was used to determine if the relative abundance was inversely correlated with the bacterial load in the DNA extract[[3](#_ENREF_3)]. Nine potential contaminant genera were detected in >20 specimens with a negative correlation (Spearman rho between -0.7 and -1.0) between the total bacterial load and the genera’s relative abundance, and were excluded from downstream analyses. These genera included *Acinetobacter*, *Stenotrophomonas* and *Burkholderia* (taxa of potential clinical significance in respiratory disease) which all showed a strong negative correlation between bacterial load and relative abundance (Additional Figure 5). Four genera reported previously as potential contaminants did not show a negative correlation between the bacterial load and the genera’s relative abundance (*Streptococcus*, *Pseudomonas*, *Enterobacter* and *Enhydrobacter*) and were not excluded from downstream analyses (Additional Figure 5).

A further 33/46 potential contaminant genera were present in <20 samples. Data for these genera were individually reviewed as a Spearman rank correlation performed with data from <20 specimens is unreliable. As all 33 of these genera were either i) only detected at <1% relative abundance or ii) only detected in specimens with low bacterial load, all were excluded from downstream analyses as probable contaminants.

Overall, 237 OTUs representing 54 genera were excluded as likely contaminants (including those detected in the negative control). A complete list of all contaminant genera and OTUs excluded from downstream analyses is presented in Additional Table 1.

***Additional References***

1. Dowd SE, Sun Y, Wolcott RD, Domingo A, Carroll JA. Bacterial tag-encoded FLX amplicon pyrosequencing (bTEFAP) for microbiome studies: bacterial diversity in the ileum of newly weaned *Salmonella*-infected pigs. Foodborne Pathog Dis. 2008;5(4):459-72.

2. Salter SJ, Cox MJ, Turek EM, Calus ST, Cookson WO, Moffatt MF et al. Reagent and laboratory contamination can critically impact sequence-based microbiome analyses. BMC Biol. 2014;12:87.

3. Jervis-Bardy J, Leong LE, Marri S, Smith RJ, Choo JM, Smith-Vaughan HC et al. Deriving accurate microbiota profiles from human samples with low bacterial content through post-sequencing processing of Illumina MiSeq data. Microbiome. 2015;3:19.

**Additional Figure 1: Relative abundance of contaminant OTUs in different specimen types.** Relative abundance of contaminant OTUs was significantly lower in OP swabs compared to all other specimen types (all Dunn’s *post hoc* test p<0.0001); but did not differ significantly between NP, Lavage-1 and Lavage-2 specimens. NP = NP swab, OP = OP swab, L1 = Lavage-1, L2 = Lavage-2.


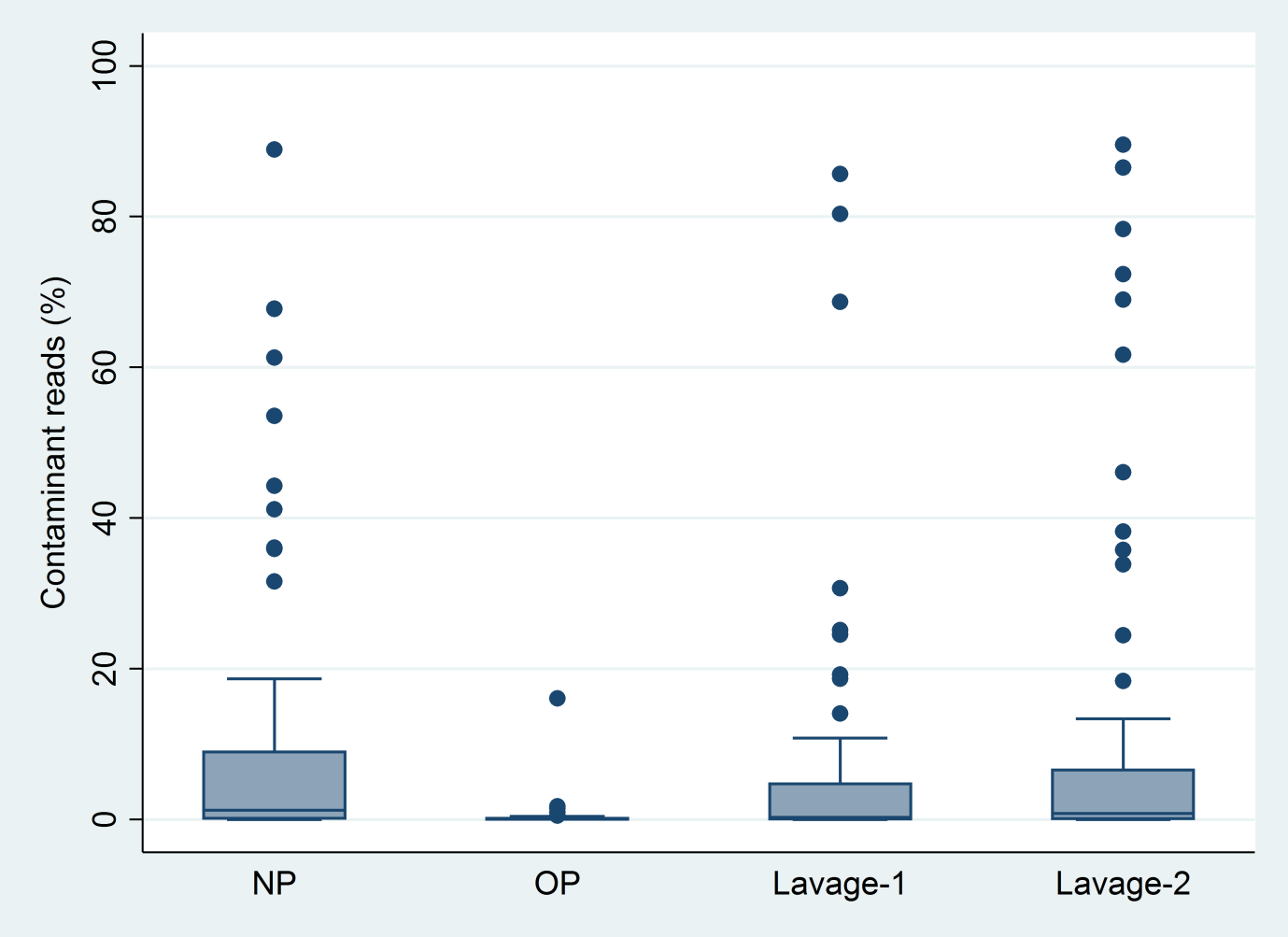


**Additional Figure 2: Principal coordinate analysis demonstrating that specimens with low bacterial load did not cluster discretely from those with >10^3^ GE/µL extracted DNA.**

This principal coordinate analysis is based on the Bray-Curtis similarity matrix derived from square root transformed OTU-level data after removal of contaminant OTUs and subsampling to 1025 reads. Data points indicate individual specimens with low bacterial load (red triangles, pointing down) and those with >10^3^ GE/µL extracted DNA (blue triangles, pointing up). Separate and discrete clustering based on bacterial load was not evident.

**
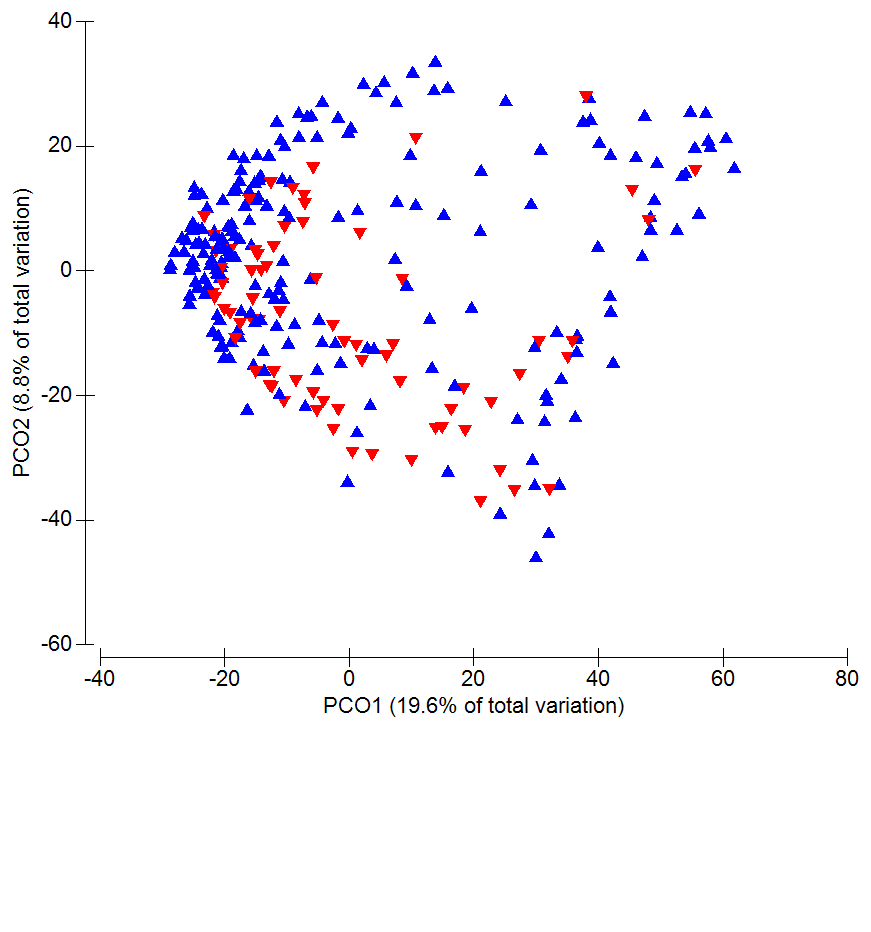
**

**Additional Figure 3: Bacterial diversity in each specimen type separated by diagnostic group.** A) Diversity in NP swabs. B) Diversity in OP swabs. C) Diversity in Lavage-1 samples. D) Diversity in Lavage-2 samples. Diversity was determined using Simpson’s Index of Diversity (1-D). Diversity approaching 1 indicates more rich and even communities. Diversity approaching zero indicates communities dominated by a small number of taxa. Diversity in NP swabs trended higher with increased disease severity; however, only the data from control children and those with CSLD were statistically different after correction for multiple methods (Dunn’s *post hoc* test p<0.0001). There were no significant differences in diversity of other specimen types between diagnostic groups.


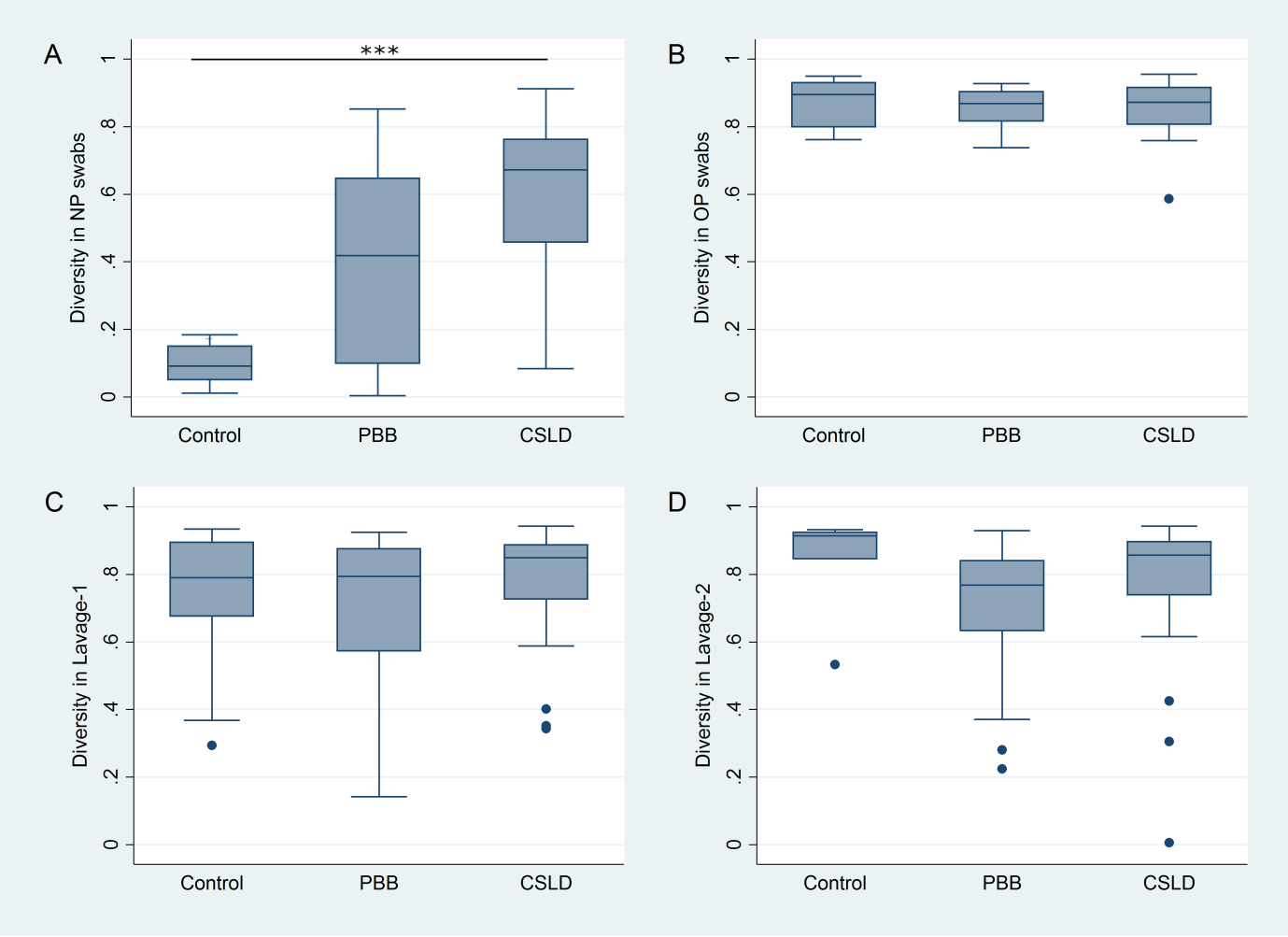


**Additional Figure 4: Similarity of OP swab and Lavage-1 microbiota.**

Hierarchical group-average cluster analysis prepared using the Bray-Curtis similarity matrix based on square-root transformed OTU-level data. Coloured symbols on the right of the figure indicate specimens from individual children. A similarity profile permutation test was used to identify clusters with no significant dissimilarity (red dashed branches). Paired data points positioned side-by-side at the end of red branches showed no significant dissimilarity. The dashed black line indicates 50% similarity in the cluster analysis. Paired OP swab and Lavage-1 specimens from 27/65 clustered together without significant dissimilarity.


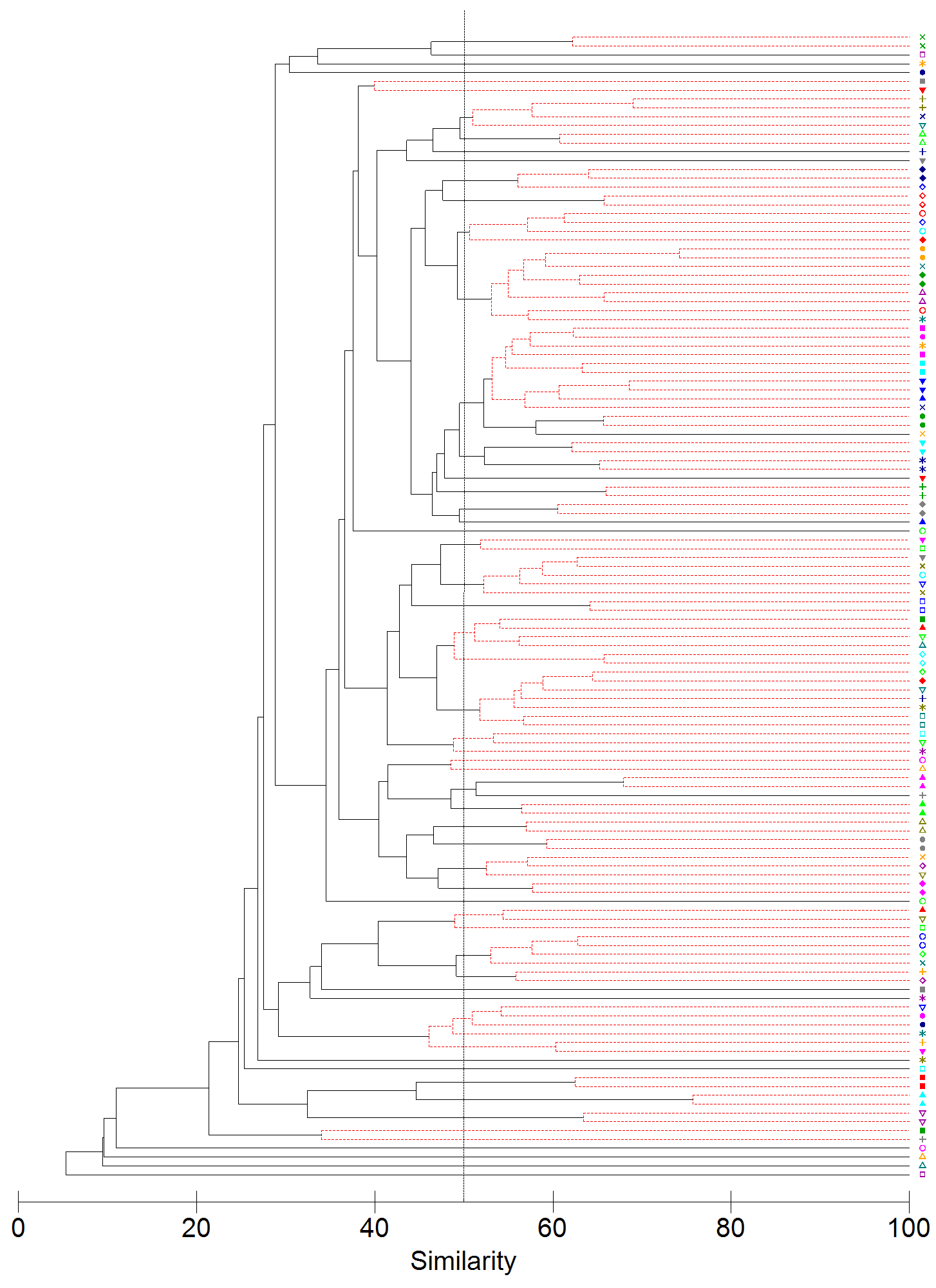


**Additional Figure 5:** Scatterplots representative of relationships observed for potential contaminant taxa in clinical specimens. Spearman’s correlation coefficient (rho) and p-values are shown for taxa that showed a significant inverse correlation between the taxa’s relative abundance and the bacterial load in the DNA extract. OTU00003 was detected in 121 samples, including 1/2 negative controls from sequencing batch 2, but did not show an inverse relationship between its relative abundance and the total bacterial load.


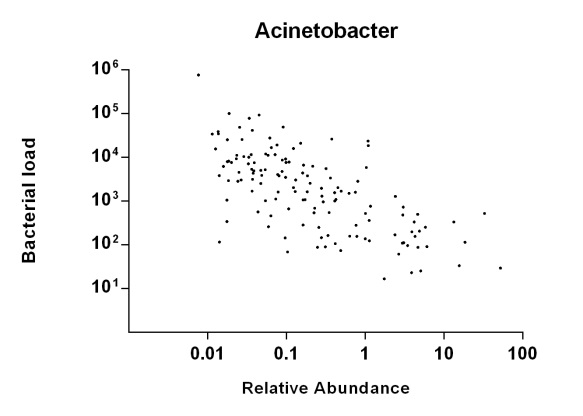

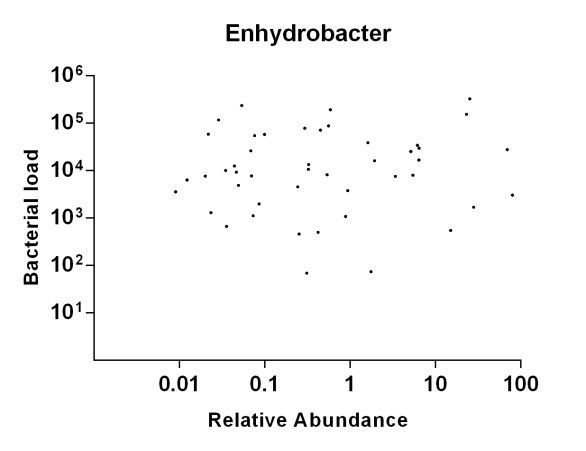

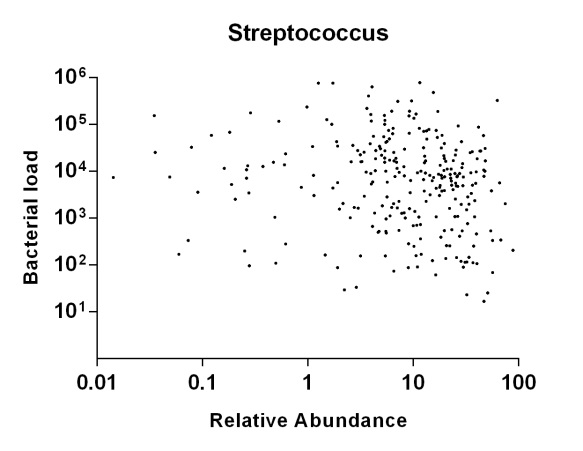

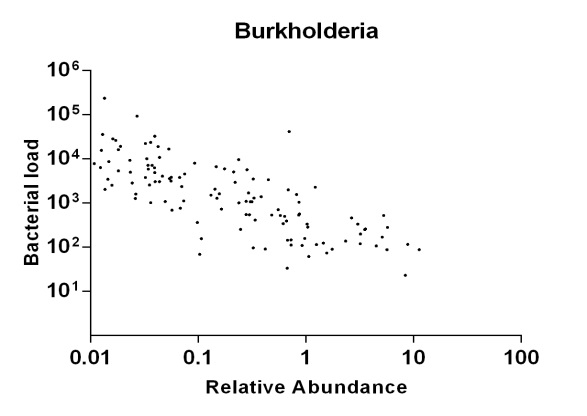

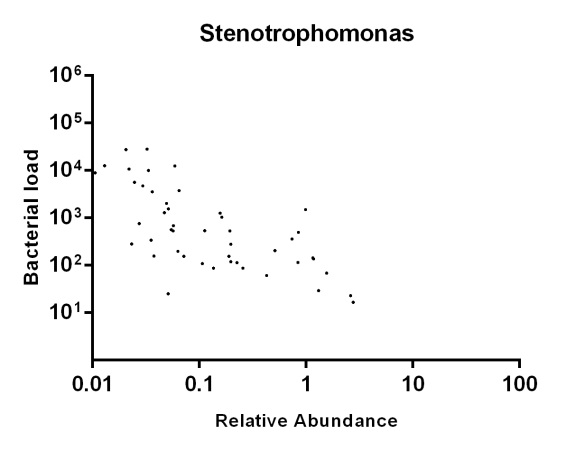

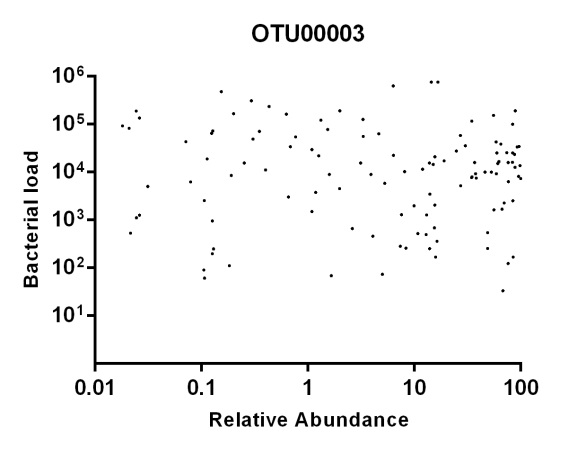


rho -0.7464

p<0.0001

rho -0.8510

p<0.0001

rho -0.7527

p<0.0001

**Additional Table 1:** **Probable contaminant OTUs removed prior to downstream analyses.**

Probable contaminant genera were identified based on an inverse Spearman correlation between the total bacterial load and the relative abundance of each genera in the sequencing data. Twenty-three of the 237 OTUs identified as probable contaminants were also present in at least of the DNA extraction negative controls.

| Genus | OTU number | Was the contaminant detected in a DNA extraction negative control? |
| --- | --- | --- |
| Abiotrophia | Otu00117 | no |
| Abiotrophia | Otu01298 | no |
| Abiotrophia | Otu01645 | no |
| Abiotrophia | Otu02348 | no |
| Abiotrophia | Otu02495 | no |
| Abiotrophia | Otu02528 | no |
| Abiotrophia | Otu02813 | no |
| Abiotrophia | Otu03394 | no |
| Abiotrophia | Otu03508 | no |
| Achromobacter | Otu00225 | yes |
| Acidovorax | Otu00393 | no |
| Acidovorax | Otu00408 | yes |
| Acinetobacter | Otu00026 | yes |
| Acinetobacter | Otu00132 | no |
| Acinetobacter | Otu00139 | no |
| Acinetobacter | Otu00195 | no |
| Acinetobacter | Otu00196 | no |
| Acinetobacter | Otu00346 | yes |
| Acinetobacter | Otu00358 | no |
| Acinetobacter | Otu00365 | no |
| Acinetobacter | Otu00390 | no |
| Acinetobacter | Otu00584 | no |
| Acinetobacter | Otu00694 | no |
| Acinetobacter | Otu00697 | no |
| Acinetobacter | Otu00823 | no |
| Acinetobacter | Otu01108 | no |
| Acinetobacter | Otu01180 | no |
| Acinetobacter | Otu01310 | no |
| Acinetobacter | Otu01339 | no |
| Acinetobacter | Otu02045 | no |
| Acinetobacter | Otu02640 | no |
| Acinetobacter | Otu02724 | no |
| Acinetobacter | Otu02743 | no |
| Acinetobacter | Otu02820 | no |
| Acinetobacter | Otu02972 | no |
| Acinetobacter | Otu03325 | no |
| Acinetobacter | Otu03636 | no |
| Acinetobacter | Otu03666 | no |
| Acinetobacter | Otu03719 | no |
| Acinetobacter | Otu03721 | no |
| Acinetobacter | Otu03825 | no |
| Acinetobacter | Otu03956 | no |
| Acinetobacter | Otu03976 | no |
| Acinetobacter | Otu04164 | no |
| Acinetobacter | Otu04190 | no |
| Acinetobacter | Otu00199 | no |
| Acinomycetales | Otu00884 | yes |
| Acintinomycetales | Otu00313 | yes |
| Aeromicrobium | Otu00565 | no |
| Aeromicrobium | Otu01174 | no |
| Anoxybacillus | Otu00701 | yes |
| Aquabacterium | Otu02061 | no |
| Arthrobacter | Otu01663 | no |
| Arthrobacter | Otu00536 | no |
| Arthrobacter | Otu00320 | no |
| Arthrobacter | Otu02797 | no |
| Arthrobacter | Otu00642 | no |
| Arthrobacter | Otu00614 | no |
| Bacillus | Otu00213 | no |
| Bacillus | Otu00298 | no |
| Bacillus | Otu01055 | no |
| Bacillus | Otu01198 | no |
| Bacillus | Otu01658 | no |
| Bacillus | Otu04070 | no |
| Bosea | Otu00913 | no |
| Bosea | Otu01916 | no |
| Bosea | Otu02112 | no |
| Bradyrhizobium | Otu00264 | yes |
| Bradyrhizobium | Otu01573 | no |
| Brevibacterium | Otu00691 | no |
| Brevibacterium | Otu01277 | no |
| Brevibacterium | Otu01364 | no |
| Brevibacterium | Otu01404 | no |
| Brevundimonas | Otu00303 | no |
| Brevundimonas | Otu00698 | no |
| Brevundimonas | Otu02163 | no |
| Burkholderia | Otu00007 | yes |
| Burkholderia | Otu00249 | no |
| Burkholderia | Otu00957 | no |
| Burkholderia | Otu01432 | no |
| Burkholderia | Otu01507 | no |
| Burkholderia | Otu03041 | no |
| Burkholderia | Otu03388 | no |
| Burkholderia | Otu03389 | no |
| Burkholderia | Otu03417 | no |
| Burkholderia | Otu03466 | no |
| Burkholderia | Otu03527 | no |
| Burkholderia | Otu03921 | no |
| Burkholderia | Otu04267 | no |
| Caulobacter | Otu00531 | no |
| Chryseobacterium | Otu00789 | no |
| Chryseobacterium | Otu01718 | no |
| Chryseobacterium | Otu02646 | no |
| Chryseobacterium | Otu03194 | no |
| Chryseobacterium | Otu04197 | no |
| Cloacibacterium | Otu00082 | yes |
| Comamonadaceae | Otu02293 | yes |
| Comamonas | Otu00451 | no |
| Comamonas | Otu00635 | no |
| Comamonas | Otu02285 | no |
| Comamonas | Otu02432 | no |
| Comamonas | Otu02526 | no |
| Comamonas | Otu02612 | no |
| Corynebacterium | Otu00110 | yes |
| Cupriavidus | Otu01883 | no |
| Devosia | Otu01187 | no |
| Devosia | Otu02268 | no |
| Duganella | Otu00171 | no |
| Enterobacter | Otu00189 | yes |
| Escherichia | Otu03583 | no |
| Flavobacterium | Otu00153 | no |
| Flavobacterium | Otu00236 | no |
| Flavobacterium | Otu00291 | no |
| Flavobacterium | Otu00297 | no |
| Flavobacterium | Otu00415 | no |
| Flavobacterium | Otu00417 | no |
| Flavobacterium | Otu00617 | no |
| Flavobacterium | Otu00632 | no |
| Flavobacterium | Otu00636 | no |
| Flavobacterium | Otu00640 | no |
| Flavobacterium | Otu00708 | no |
| Flavobacterium | Otu00782 | no |
| Flavobacterium | Otu00839 | no |
| Flavobacterium | Otu00881 | no |
| Flavobacterium | Otu01077 | no |
| Flavobacterium | Otu01100 | no |
| Flavobacterium | Otu01210 | no |
| Flavobacterium | Otu01265 | no |
| Flavobacterium | Otu01269 | no |
| Flavobacterium | Otu01496 | no |
| Flavobacterium | Otu01641 | no |
| Flavobacterium | Otu01670 | no |
| Flavobacterium | Otu01751 | no |
| Flavobacterium | Otu01786 | no |
| Flavobacterium | Otu01895 | no |
| Flavobacterium | Otu01975 | no |
| Flavobacterium | Otu02090 | no |
| Flavobacterium | Otu02266 | no |
| Flavobacterium | Otu02452 | no |
| Flavobacterium | Otu02759 | no |
| Flavobacterium | Otu02760 | no |
| Flavobacterium | Otu03033 | no |
| Flavobacterium | Otu03909 | no |
| Herbaspirillum | Otu00845 | no |
| Janthinobacterium | Otu00119 | no |
| Janthinobacterium | Otu01691 | no |
| Kingella | Otu00429 | no |
| Kingella | Otu01002 | no |
| Limnobacter | Otu03514 | no |
| Massilia | Otu00186 | no |
| Massilia | Otu00391 | no |
| Massilia | Otu00397 | no |
| Massilia | Otu00456 | no |
| Massilia | Otu00464 | no |
| Massilia | Otu00488 | no |
| Massilia | Otu00511 | no |
| Massilia | Otu00948 | no |
| Massilia | Otu01424 | no |
| Massilia | Otu02320 | no |
| Massilia | Otu03981 | no |
| Massilia | Otu00732 | no |
| Massilia | Otu00367 | no |
| Massilia | Otu00237 | no |
| Methylophilus | Otu00370 | no |
| Methylophilus | Otu00763 | no |
| Methyloversatilis | Otu01341 | no |
| Nevskia | Otu03996 | no |
| Novosphingobium | Otu00546 | no |
| Novosphingobium | Otu00606 | no |
| Novosphingobium | Otu00653 | no |
| Novosphingobium | Otu00872 | no |
| Novosphingobium | Otu01127 | no |
| Novosphingobium | Otu01860 | no |
| Novosphingobium | Otu03884 | no |
| Paracoccus | Otu00939 | no |
| Paracoccus | Otu01120 | no |
| Paracoccus | Otu01383 | no |
| Pedobacter | Otu00907 | no |
| Pedobacter | Otu00984 | no |
| Pedobacter | Otu01208 | no |
| Pedobacter | Otu01743 | no |
| Pedobacter | Otu02072 | no |
| Pedobacter | Otu02080 | no |
| Pedobacter | Otu02303 | no |
| Pelomonas | Otu00228 | no |
| Pelomonas | Otu01618 | no |
| Polaromonas | Otu00169 | no |
| Polaromonas | Otu00175 | no |
| Polaromonas | Otu00316 | no |
| Polaromonas | Otu01255 | no |
| Polaromonas | Otu01857 | no |
| Polaromonas | Otu01272 | no |
| Polaromonas | Otu00311 | no |
| Propionibacterium | Otu01061 | yes |
| Propionibacterium | Otu03221 | yes |
| Propionibacterium | Otu00063 | yes |
| Pseudomonas | Otu00157 | yes |
| Pseudomonas | Otu00187 | yes |
| Pseudoxanthomonas | Otu00484 | no |
| Psychrobacter | Otu02398 | no |
| Ralstonia | Otu00033 | yes |
| Ralstonia | Otu01993 | no |
| Ralstonia | Otu02782 | no |
| Ralstonia | Otu02911 | no |
| Ralstonia | Otu03572 | no |
| Ralstonia | Otu03695 | no |
| Schlegelella | Otu00929 | no |
| Serratia | Otu00142 | yes |
| Sphingobium | Otu00212 | no |
| Sphingobium | Otu02070 | no |
| Sphingomonas | Otu00767 | no |
| Sphingomonas | Otu00781 | no |
| Sphingomonas | Otu00793 | no |
| Sphingomonas | Otu00854 | no |
| Sphingomonas | Otu01020 | no |
| Sphingomonas | Otu01473 | no |
| Sphingomonas | Otu01831 | no |
| Sphingomonas | Otu01854 | no |
| Sphingomonas | Otu01874 | no |
| Sphingomonas | Otu02027 | no |
| Sphingomonas | Otu02077 | no |
| Sphingomonas | Otu02078 | no |
| Sphingomonas | Otu02294 | no |
| Sphingomonas | Otu03886 | no |
| Sphingomonas | Otu03944 | no |
| Sphingomonas | Otu01096 | no |
| Sphingomonas | Otu00595 | no |
| Staphylococcus | Otu02663 | yes |
| Staphylococcus | Otu00041 | yes |
| Stenotrophomonas | Otu00070 | yes |
| Stenotrophomonas | Otu00523 | no |
| Stenotrophomonas | Otu02864 | no |
| Stenotrophomonas | Otu01089 | no |
| Undibacterium | Otu00922 | no |
| Undibacterium | Otu01638 | no |
| Variovorax | Otu00154 | no |
| Variovorax | Otu02380 | no |
